# Supplementary material for: Impact of noradrenergic inhibition on neuroinflammation and pathophysiology in mouse models of Alzheimer’s disease
Source: J Neuroinflammation. 2024 Dec 18;21:322. doi: 10.1186/s12974-024-03306-1 (PMC11657531; doi:10.1186/s12974-024-03306-1)
Supplement: Supplementary file 1 — Supplementary Material 1 [file 12974_2024_3306_MOESM1_ESM.pdf]

Supplementary Tables

Supplementary Table S1. Luminex Cytokine Data - ANOVA Table for 5XFAD DREADD – female, brain homogenate.

Supplemental Table 1. ANOVA Table for 5XFAD DREADD.

	FEMALE DREADD (two-way ANOVA effects of 5XFAD x DREADD)						Sidak's			
MARKER	INTERACTION		5XFAD		DREADD		NC-con v 5x-con	NC-DREADD v 5X-DREADD	NC-con v NC-DREADD	5X-con v 5X-DREADD
BAFF	F (1, 38) = 4.036	(trend) P=0.0517	F (1, 38) = 167.8	*** P<0.0001	F (1, 38) = 5.552	* P=0.0237	*** 0.0001	*** 0.0001	0.9981	* 0.0253
GCSF	F (1, 39) = 0.07256	P=0.7891	F (1, 39) = 0.4024	P=0.5296	F (1, 39) = 0.4712	P=0.4965	0.9961	0.9706	0.9229	0.9978
GMCSF	F (1, 39) = 1.342	P=0.2538	F (1, 39) = 1.039	P=0.3144	F (1, 39) = 0.0001146	P=0.9915	0.229	0.9999	0.8531	0.9084
IFNA	F (1, 39) = 0.2314	P=0.6332	F (1, 39) = 3.229	(trend) P=0.0801	F (1, 39) = 0.5152	P=0.4772	0.6908	0.5345	0.8415	0.9998
IFNG	F (1, 38) = 1.882	P=0.1781	F (1, 38) = 1.291	P=0.2630	F (1, 38) = 0.1645	P=0.6874	0.9993	0.4346	0.5536	0.9497
IL1A	F (1, 39) = 0.4906	P=0.4878	F (1, 39) = 9.503	** P=0.0038	F (1, 39) = 1.831e-005	P=0.9966	0.1604	0.1034	0.9734	0.9843
IL1B	F (1, 39) = 0.001808	P=0.9663	F (1, 39) = 1.351	P=0.2522	F (1, 39) = 1.014	P=0.3201	0.7544	0.9376	0.8951	0.949
IL2	F (1, 39) = 2.161	P=0.1496	F (1, 39) = 0.3703	P=0.5463	F (1, 39) = 0.7278	P=0.3988	0.9107	0.6181	0.3022	0.99
IL3	F (1, 39) = 0.3628	P=0.5504	F (1, 39) = 7.656	** P=0.0086	F (1, 39) = 0.2101	P=0.6492	0.2338	0.1763	0.8916	0.9999
IL4	F (1, 39) = 0.05465	P=0.8164	F (1, 39) = 5.724	* P=0.0216	F (1, 39) = 0.1578	P=0.6933	0.1012	0.5844	0.9823	0.9999
IL5	F (1, 39) = 0.2532	P=0.6176	F (1, 39) = 12.60	*** P=0.0010	F (1, 39) = 0.08007	P=0.7787	* 0.0424	(trend) 0.0711	0.9997	0.9754
IL6	F (1, 39) = 1.478	P=0.2313	F (1, 39) = 2.092	P=0.1561	F (1, 39) = 0.08861	P=0.7675	0.9994	0.3829	0.6972	0.9571
IL7	F (1, 39) = 0.4698	P=0.4971	F (1, 39) = 0.2444	P=0.6238	F (1, 39) = 1.085	P=0.3039	0.768	0.9999	0.998	0.6963
IL9	F (1, 39) = 0.001066	P=0.9741	F (1, 39) = 0.06721	P=0.7968	F (1, 39) = 0.5893	P=0.4473	0.9994	0.9996	0.9581	0.9808
IL10	F (1, 39) = 0.3541	P=0.5553	F (1, 39) = 0.2736	P=0.6039	F (1, 39) = 0.002429	P=0.9609	0.8005	0.9999	0.9808	0.9937
IL12P70	F (1, 39) = 0.2814	P=0.5988	F (1, 39) = 1.398	P=0.2442	F (1, 39) = 0.03341	P=0.8559	0.4533	0.9912	0.9982	0.9828
IL13	F (1, 39) = 0.07328	P=0.7880	F (1, 39) = 1.539	P=0.2222	F (1, 39) = 0.01390	P=0.9067	0.8695	0.8363	0.9972	0.9999
IL15/IL15R	F (1, 39) = 0.6600	P=0.4215	F (1, 39) = 11.25	** P=0.0018	F (1, 39) = 0.02626	P=0.8721	0.1194	(trend) 0.0605	0.9802	0.9472
IL17A/CTLA8	F (1, 39) = 0.1715	P=0.6810	F (1, 39) = 14.25	*** P=0.0005	F (1, 39) = 0.5483	P=0.4634	** 0.0028	0.1781	0.9986	0.9065
IL18	F (1, 39) = 0.03704	P=0.8484	F (1, 39) = 2.280	P=0.1391	F (1, 39) = 0.7299	P=0.3981	0.4594	0.893	0.9788	0.9325
IL19	F (1, 39) = 0.4328	P=0.5145	F (1, 39) = 5.661	* P=0.0223	F (1, 39) = 1.509	P=0.2266	* 0.0433	0.7624	0.5036	0.9926
IL22	F (1, 39) = 0.1357	P=0.7146	F (1, 39) = 6.840	* P=0.0126	F (1, 39) = 0.5201	P=0.4751	0.2035	0.2756	0.9981	0.9229
IL23	F (1, 39) = 0.08617	P=0.7707	F (1, 39) = 0.04397	P=0.8350	F (1, 39) = 0.1775	P=0.6758	0.9999	0.9968	0.972	0.9999
IL25/IL17	F (1, 39) = 0.6347	P=0.4304	F (1, 39) = 1.205	P=0.2791	F (1, 39) = 0.06739	P=0.7965	0.9982	0.6945	0.893	0.9941
IL27	F (1, 39) = 1.378	P=0.2475	F (1, 39) = 0.03125	P=0.8606	F (1, 39) = 0.01287	P=0.9103	0.8578	0.8841	0.8916	0.8678
IL28	F (1, 39) = 0.07613	P=0.7841	F (1, 39) = 1.588	P=0.2151	F (1, 39) = 0.03802	P=0.8464	0.558	0.9601	0.9941	0.9999
IL31	F (1, 39) = 0.002976	P=0.9568	F (1, 39) = 0.1087	P=0.7434	F (1, 39) = 0.5017	P=0.4830	0.9987	0.9989	0.9646	0.9876
IL33	F (1, 38) = 0.1378	P=0.7126	F (1, 38) = 0.5904	P=0.4470	F (1, 38) = 0.4057	P=0.5280	0.7954	0.9987	0.91	0.9996
*LIF	F (1, 39) = 0.09119	P=0.7643	F (1, 39) = 1.310	P=0.2593	F (1, 39) = 3.369	(trend) P=0.0741	0.917	0.8566	0.3815	0.7786
MCSF	F (1, 39) = 0.001835	P=0.9661	F (1, 39) = 2.799	P=0.1023	F (1, 39) = 0.1122	P=0.7394	0.5016	0.7645	0.9991	0.9985
SRANKL	F (1, 39) = 3.202	(trend) P=0.0813	F (1, 39) = 0.1468	P=0.7037	F (1, 39) = 2.220	P=0.1443	0.2308	0.8685	0.999	0.1337
TNFA	F (1, 39) = 0.9367	P=0.3391	F (1, 39) = 3.419	(trend) P=0.0720	F (1, 39) = 0.6735	P=0.4168	0.9039	0.3286	0.9999	0.6688
ENA78/LIX/CXCL5	F (1, 39) = 7.534	** P=0.0091	F (1, 39) = 8.355	** P=0.0063	F (1, 39) = 3.875	(trend) P=0.0561	0.9999	** 0.0058	0.9624	* 0.013
EOTAXIN/CCL11	F (1, 37) = 0.4295	P=0.5163	F (1, 37) = 2.636	P=0.1130	F (1, 37) = 0.01507	P=0.9030	0.174	0.9641	0.9578	0.9948
GROA/KC/CXCL1	F (1, 39) = 9.245	** P=0.0042	F (1, 39) = 11.74	** P=0.0015	F (1, 39) = 0.5104	P=0.4792	0.9952	** 0.0013	0.301	(trend) 0.0656
*IP10/CXCL10	F (1, 39) = 2.058	P=0.1593	F (1, 39) = 74.24	*** P<0.0001	F (1, 39) = 0.3503	P=0.5574	*** 0.0001	*** 0.0001	0.9498	0.5598
MCP1/CCL2	F (1, 39) = 1.072	P=0.3068	F (1, 39) = 12.92	*** P=0.0009	F (1, 39) = 0.01319	P=0.9092	0.1153	* 0.0301	0.932	0.9077
*MCP3/CCL7	F (1, 38) = 6.737	* P=0.0133	F (1, 38) = 59.32	*** P<0.0001	F (1, 38) = 4.829	* P=0.0342	*** 0.0003	*** 0.0001	0.997	* 0.0109
*MIP1A/CCL3	F (1, 38) = 10.02	** P=0.0030	F (1, 38) = 814.4	*** P<0.0001	F (1, 38) = 11.33	** P=0.0018	*** 0.0001	*** 0.0001	0.9998	*** 0.0006
*MIP1B/CCL4	F (1, 38) = 10.66	** P=0.0023	F (1, 38) = 129.3	*** P<0.0001	F (1, 38) = 10.51	** P=0.0025	*** 0.0001	*** 0.0001	0.9999	*** 0.0005
MIP2	F (1, 39) = 2.914	(trend) P=0.0958	F (1, 39) = 2.191	P=0.1469	F (1, 39) = 0.003263	P=0.9547	0.9994	0.2191	0.6269	0.6798
*RANTES/CCL5	F (1, 38) = 4.978	* P=0.0316	F (1, 38) = 71.56	*** P<0.0001	F (1, 38) = 3.907	(trend) P=0.0554	*** 0.0001	*** 0.0001	0.9995	* 0.0274
BTC	F (1, 38) = 0.4365	P=0.5128	F (1, 38) = 0.1771	P=0.6762	F (1, 38) = 0.4834	P=0.4911	0.8268	0.9998	0.9999	0.8478
LEPTIN	F (1, 39) = 0.1729	P=0.6798	F (1, 39) = 0.7770	P=0.3835	F (1, 39) = 0.1301	P=0.7202	0.7018	0.9976	0.9623	0.9999
VEGF	F (1, 39) = 0.003983	P=0.9500	F (1, 39) = 8.830	** P=0.0051	F (1, 39) = 0.009530	P=0.9227	* 0.0435	0.2987	0.9999	0.9999
IL2RA	F (1, 39) = 0.1255	P=0.7251	F (1, 39) = 3.908	(trend) P=0.0552	F (1, 39) = 0.1376	P=0.7127	0.506	0.5125	0.9705	0.9999
IL7RA	F (1, 39) = 0.1088	P=0.7432	F (1, 39) = 1.110	P=0.2986	F (1, 39) = 0.1088	P=0.7432	0.9505	0.8751	0.9999	0.9871
ST2/IL33R	F (1, 38) = 1.390	P=0.2457	F (1, 38) = 6.922	* P=0.0122	F (1, 38) = 1.796	P=0.1881	** 0.0061	0.8592	0.2528	0.9999

Supplementary Table S2. Luminex Cytokine Data - ANOVA Table for 5XFAD Propranolol – male, brain homogenate.

Supplemental Table S2. ANOVA Table for 5XFAD Propranolol - cortical homogenate.				
			posthoc comparisons (Sidak's)	
MARKER	one-way ANOVA		male 5XFAD-veh vs NC-veh	male 5XFAD-Prop vs 5XFAD-veh
**BAFF**	**F (2, 11) = 16.09**	***** P=0.0005**	**** 0.0049**	
**GCSF**	F (2, 11) = 3.227	P=0.0789		
**GMCSF**	**F (2, 12) = 4.267**	*** P=0.0398**	ns	ns
**IFNA**	F (2, 12) = 2.667	P=0.1101		
**IFNG**	F (2, 12) = 2.660	P=0.1106		
**IL1A**	**F (2, 12) = 13.31**	***** P=0.0009**	**** 0.0018**	
**IL1B**	F (2, 12) = 2.357	P=0.1369		
**IL2**	F (2, 12) = 0.5351	P=0.5990		
**IL3**	**F (2, 12) = 11.60**	**** P=0.0016**	*** 0.0183**	
**IL4**	**F (2, 12) = 5.180**	*** P=0.0239**	*** 0.0497**	
**IL5**	F (2, 12) = 1.309	P=0.3059		
**IL6**	**F (2, 12) = 5.909**	*** P=0.0164**		*** 0.0269**
**IL7**	F (2, 12) = 2.022	P=0.1751		
**IL9**	F (2, 11) = 0.3676	P=0.7006		
**IL10**	F (2, 12) = 0.3332	P=0.7231		
**IL12P70**	F (2, 12) = 1.625	P=0.2375		
**IL13**	**F (2, 12) = 4.804**	*** P=0.0293**	(trend) 0.0772	
**IL15/IL15R**	F (2, 12) = 0.8245	P=0.4618		
**IL17A/CTLA8**	**F (2, 12) = 4.610**	*** P=0.0327**	*** 0.0481**	
**IL18**	**F (2, 12) = 11.93**	**** P=0.0014**	**** 0.0043**	
**IL19**	F (2, 12) = 0.1844	P=0.8339		
**IL22**	F (2, 12) = 3.704	(trend) P=0.0559	ns	ns
**IL23**	F (2, 11) = 1.859	P=0.2016		
**IL25/IL17**	F (2, 12) = 2.525	P=0.1216		
**IL27**	F (2, 12) = 2.541	P=0.1202		
**IL28**	F (2, 12) = 0.4074	P=0.6743		
**IL31**	F (2, 12) = 1.705	P=0.2231		
**IL33**	F (2, 12) = 2.575	P=0.1174		
**LIF**	**F (2, 12) = 9.315**	**** P=0.0036**		(trend) 0.0685
**MCSF**	**F (2, 11) = 5.825**	*** P=0.0188**	(trend) 0.0971	
**SRANKL**	F (2, 12) = 3.675	(trend) P=0.0569	ns	ns
**TNFA**	F (2, 11) = 1.240	P=0.3269		
**ENA78/LIX/CXCL5**	F (2, 11) = 1.502	P=0.2650		
**EOTAXIN/CCL11**	**F (2, 11) = 62.11**	***** P<0.0001**		***** 0.0001**
**GROA/KC/CXCL1**	**F (2, 11) = 8.318**	**** P=0.0063**		*** 0.0177**
***IP10/CXCL10**	**F (2, 12) = 5.892**	*** P=0.0165**	*** 0.0155**	
**MCP1/CCL2**	F (2, 10) = 1.720	P=0.2280		
***MCP3/CCL7**	**F (2, 12) = 5.783**	*** P=0.0174**		(trend) 0.0820
***MIP1A/CCL3**	**F (2, 12) = 11.07**	**** P=0.0019**	**** 0.0035**	
***MIP1B/CCL4**	**F (2, 12) = 5.374**	*** P=0.0216**	*** 0.050**	
**MIP2**	**F (2, 11) = 22.71**	***** P=0.0001**	***** 0.0008**	
***RANTES/CCL5**	**F (2, 12) = 4.846**	*** P=0.0287**	(trend) 0.0645	
**BTC**	F (2, 12) = 0.5796	P=0.5750		
**LEPTIN**	F (2, 12) = 3.148	(trend) P=0.0796	ns	ns
**VEGF**	F (2, 11) = 1.444	P=0.2774		
**IL2RA**	**F (2, 11) = 5.514**	*** P=0.0219**	ns	ns
**IL7RA**	F (2, 12) = 1.490	P=0.2643		
**ST2/IL33R**	F (2, 12) = 3.008	(trend) P=0.0873	ns	ns

Supplementary Table S3. Luminex Cytokine Data - ANOVA Table for 5XFAD Propranolol – male, plasma.

**Supplemental Table S3. ANOVA Table for 5XFAD Propranolol - plasma.**				
			posthoc comparisons (Sidak's)	
**MARKER**	**one-way ANOVA**		**male 5XFAD-veh vs NC-veh**	**male 5XFAD-Prop vs 5XFAD-veh**
**BAFF**	F (2, 11) = 1.764	P=0.2166		
**GCSF**	**F (2, 11) = 5.594**	**P=0.0211**		*** 0.0306**
**GMCSF**	F (2, 11) = 1.660	P=0.2345		
**IFNA**	F (2, 10) = 2.093	P=0.1741		
**IFNG**	F (2, 10) = 3.394	(trend) P=0.0750		
**IL1A**	F (2, 10) = 1.916	P=0.1975		
**IL1B**	F (2, 11) = 0.1331	P=0.8768		
**IL2**	F (2, 10) = 0.1300	P=0.8796		
**IL3**	F (2, 10) = 2.611	P=0.1223		
**IL4**	**F (2, 9) = 8.003**	*** P=0.0101**	ns	ns
**IL5**	F (2, 11) = 0.03793	P=0.9629		
**IL6**	F (2, 11) = 3.244	(trend) P=0.0781	ns	ns
**IL7**	F (2, 9) = 0.4150	P=0.6724		
**IL9**	F (2, 10) = 3.207	(trend) P=0.0839	(trend) 0.0693	
**IL10**	F (2, 10) = 0.7616	P=0.4922		
**IL12P70**	F (2, 10) = 2.187	P=0.1630		
**IL13**	F (2, 9) = 1.191	P=0.3476		
**IL15/IL15R**	F (2, 10) = 0.1760	P=0.8412		
**IL17A/CTLA8**	F (2, 10) = 2.068	P=0.1771		
**IL18**	F (2, 11) = 0.4257	P=0.6636		
**IL19**	F (2, 10) = 0.5443	P=0.5965		
**IL22**	F (2, 11) = 2.299	P=0.1465		
**IL23**	F (2, 10) = 0.3456	P=0.7159		
**IL25/IL17**	F (2, 10) = 0.1841	P=0.8346		
**IL27**	F (2, 9) = 0.8193	P=0.4711		
**IL28**	F (2, 11) = 0.5463	P=0.5940		
**IL31**	F (2, 11) = 0.1652	P=0.8498		
**IL33**	F (2, 10) = 0.5842	P=0.5755		
**LIF**	F (2, 11) = 1.496	P=0.2662		
**MCSF**	F (2, 9) = 1.285	P=0.3229		
**SRANKL**	F (2, 9) = 0.2306	P=0.7986		
**TNFA**	F (2, 11) = 1.210	P=0.3351		
**ENA78/LIX/CXCL5**	F (2, 9) = 0.9530	P=0.4213		
**EOTAXIN/CCL11**	**F (2, 11) = 6.499**	*** P=0.0137**	ns	ns
**GROA/KC/CXCL1**	F (2, 11) = 3.847	(trend) P=0.0541		*** 0.0413**
**IP10/CXCL10**	F (2, 11) = 3.007	(trend) P=0.0908		
**MCP1/CCL2**	F (2, 10) = 2.730	P=0.1132		
***MCP3/CCL7**	**F (2, 11) = 5.581**	*** P=0.0212**		*** 0.0251**
***MIP1A/CCL3**	F (2, 11) = 1.834	P=0.2055		
***MIP1B/CCL4**	**F (2, 11) = 8.239**	**** P=0.0065**	ns	ns
**MIP2**	**F (2, 9) = 7.875**	*** P=0.0105**	(trend) 0.0535	
***RANTES/CCL5**	F (2, 9) = 2.973	P=0.1021	(trend) 0.0973	
**BTC**	F (2, 11) = 0.7872	P=0.4791		
**LEPTIN**	**F (2, 9) = 6.311**	*** P=0.0194**	*** 0.0497**	*** 0.0140**
**VEGF**	F (2, 11) = 0.5409	P=0.5969		
**IL2RA**	F (2, 11) = 0.9915	P=0.4019		
**IL7RA**	F (2, 10) = 3.172	(trend) P=0.0857		
**ST2/IL33R**	F (2, 10) = 4.031	(trend) P=0.0520		(trend) 0.0852

Supplementary Table S4. Luminex Cytokine Data - ANOVA Table for 5XFAD adrb2 cKO – male, brain homogenate.

**Supplemental Table S4 - Luminex Data ANOVA Table for ADRB2 conditional KO - male data**										
	MALE ADRB2 cKO (two-way ANOVA effects of GENE x cKO)						Sidak's			
	**INTERACTION**		**GENE**		**cKO**		**wt-con v 5x-con**	**wt-cKO v 5X-cKO**	**wt-con v wt-cKO**	**5X-con v 5X cKO**
**BAFF**	F (1, 35) = 0.1392	P=0.7113	**F (1, 35) = 68.35**	***** P<0.0001**	F (1, 35) = 0.8510	P=0.3626	***** <0.0001**	***** <0.0001**	0.8447	0.9915
**GCSF**	F (1, 34) = 1.819	P=0.1863	F (1, 34) = 1.697	P=0.2014	F (1, 34) = 0.006124	P=0.9381	0.2274	0.9999	0.7863	0.8477
**GMCSF**	F (1, 35) = 0.2760	P=0.6026	**F (1, 35) = 4.450**	*** P=0.0421**	F (1, 35) = 0.3523	P=0.5566	0.243	0.7261	0.9999	0.8929
**IFNA**	F (1, 35) = 0.4035	P=0.5294	F (1, 35) = 0.8701	P=0.3573	F (1, 35) = 0.02387	P=0.8781	0.9992	0.7334	0.9704	0.9949
**IFNG**	F (1, 34) = 0.8716	P=0.3571	F (1, 34) = 0.004477	P=0.9470	F (1, 34) = 0.02546	P=0.8742	0.9292	0.9568	0.9711	0.905
**IL1A**	F (1, 34) = 0.02435	P=0.8769	F (1, 34) = 0.08350	P=0.7744	F (1, 34) = 0.7818	P=0.3828	0.9964	0.9999	0.9259	0.9745
**IL1B**	F (1, 35) = 0.06578	P=0.7991	**F (1, 35) = 8.597**	**** P=0.0059**	F (1, 35) = 1.705	P=0.2002	0.2302	0.1243	0.736	0.9131
**IL2**	F (1, 35) = 0.006066	P=0.9384	F (1, 35) = 0.0006740	P=0.9794	F (1, 35) = 0.03303	P=0.8568	0.9999	0.9999	0.9996	0.9999
**IL3**	F (1, 34) = 2.066	P=0.1597	F (1, 34) = 2.066	P=0.1597	F (1, 34) = 0.4016	P=0.5305	0.1658	0.9999	0.9669	0.4834
**IL4**	F (1, 34) = 1.141	P=0.2929	F (1, 34) = 3.000	P=0.0923	F (1, 34) = 1.364	P=0.2510	0.1847	0.985	0.4086	0.9999
**IL5**	F (1, 35) = 0.6422	P=0.4283	F (1, 35) = 1.794	P=0.1890	F (1, 35) = 0.2737	P=0.6042	0.9921	0.4636	0.9995	0.8202
**IL6**	F (1, 33) = 0.4877	P=0.4899	F (1, 33) = 0.8114	P=0.3742	F (1, 33) = 0.04404	P=0.8351	0.6981	0.9998	0.9519	0.9945
**IL7**	F (1, 35) = 0.3107	P=0.5808	F (1, 35) = 4.979e-00	P=0.9944	F (1, 35) = 0.001792	P=0.9665	0.9914	0.9915	0.994	0.9881
**IL9**	F (1, 34) = 0.0007031	P=0.9790	F (1, 34) = 2.482	P=0.1244	F (1, 34) = 0.9994	P=0.3245	0.7085	0.7329	0.9292	0.9293
**IL10**	**F (1, 34) = 3.294**	**P=0.0784 (trend)**	F (1, 34) = 0.08340	P=0.7745	F (1, 34) = 1.797	P=0.1889	0.4387	0.7622	0.9959	0.1063
**IL12P70**	F (1, 35) = 2.277	P=0.1403	F (1, 35) = 0.1247	P=0.7261	F (1, 35) = 0.009487	P=0.9230	0.5707	0.891	0.716	0.7843
**IL13**	F (1, 35) = 0.3860	P=0.5384	F (1, 35) = 0.1199	P=0.7312	F (1, 35) = 0.3673	P=0.5484	0.9994	0.9395	0.9999	0.8569
**IL15/IL15R**	F (1, 35) = 0.2988	P=0.5881	F (1, 35) = 0.2346	P=0.6311	F (1, 35) = 0.06516	P=0.8000	0.9179	0.9999	0.9687	0.9993
**IL17A/CTLA8**	F (1, 34) = 0.03408	P=0.8546	F (1, 34) = 1.943	P=0.1723	F (1, 34) = 2.576	P=0.1177	0.6967	0.8802	0.8055	0.5921
**IL18**	F (1, 35) = 0.02349	P=0.8791	F (1, 35) = 1.339	P=0.2551	F (1, 35) = 0.8315	P=0.3681	0.8258	0.9314	0.9164	0.9717
**IL19**	F (1, 35) = 0.0007972	P=0.9776	F (1, 35) = 4.414	P=0.0429	F (1, 35) = 1.777	P=0.1911	0.4426	0.495	0.82	0.828
**IL22**	**F (1, 34) = 5.320**	*** P=0.0273**	F (1, 34) = 1.860	P=0.1816	F (1, 34) = 0.9325	P=0.3410	**0.0542**	0.9421	0.1171	0.8066
**IL23**	F (1, 33) = 2.440	P=0.1278	**F (1, 33) = 3.106**	**P=0.0873 (trend)**	F (1, 33) = 0.003119	P=0.9558	0.9998	0.1022	0.7437	0.711
**IL25/IL17**	F (1, 35) = 0.3035	P=0.5852	F (1, 35) = 0.9567	P=0.3347	F (1, 35) = 0.08501	P=0.7723	0.9968	0.7506	0.9627	0.9995
**IL27**	F (1, 35) = 0.01802	P=0.8940	F (1, 35) = 2.683	P=0.1104	F (1, 35) = 0.5256	P=0.4733	0.6151	0.7617	0.9899	0.956
**IL28**	F (1, 35) = 0.9567	P=0.3347	F (1, 35) = 0.2392	P=0.6278	**F (1, 35) = 3.229**	**P=0.0810 (trend)**	0.76	0.9951	0.2223	0.9628
**IL31**	F (1, 34) = 0.4910	P=0.4882	F (1, 34) = 1.964	P=0.1701	F (1, 34) = 0.0002536	P=0.9874	0.4394	0.9819	0.9803	0.9793
**IL33**	F (1, 34) = 0.8356	P=0.3671	F (1, 34) = 0.04109	P=0.8406	F (1, 34) = 0.5677	P=0.4564	0.8982	0.9787	0.9999	0.6777
***LIF**	F (1, 34) = 0.005415	P=0.9418	**F (1, 34) = 22.04**	***** P<0.0001**	F (1, 34) = 0.3030	P=0.5856	**0.0058**	**0.0123**	0.9869	0.9953
**MCSF**	F (1, 34) = 0.1900	P=0.6657	**F (1, 34) = 6.868**	*** P=0.0130**	F (1, 34) = 0.4152	P=0.5236	0.1261	0.4565	0.9086	0.9998
**SRANKL**	F (1, 34) = 0.04875	P=0.8266	F (1, 34) = 0.9506	P=0.3364	F (1, 34) = 0.1338	P=0.7168	0.9707	0.8844	0.9999	0.9884
**TNFA**	F (1, 34) = 0.002278	P=0.9622	F (1, 34) = 1.308	P=0.2608	F (1, 34) = 0.006738	P=0.9351	0.8628	0.9128	0.9999	0.9999
**0**										
**ENA78/LIX/CXCL5**	F (1, 35) = 0.06134	P=0.8058	F (1, 35) = 1.559	P=0.2201	F (1, 35) = 0.7632	P=0.3883	0.9256	0.7648	0.9874	0.8922
**EOTAXIN/CCL11**	F (1, 35) = 0.1134	P=0.7383	F (1, 35) = 0.7567	P=0.3903	F (1, 35) = 2.183	P=0.1485	0.9924	0.8751	0.8955	0.5943
**GROA/KC/CXCL1**	F (1, 35) = 0.6658	P=0.4200	F (1, 35) = 0.9317	P=0.3411	F (1, 35) = 0.9942	P=0.3256	0.6106	0.9999	0.6187	0.9999
***IP10/CXCL10**	F (1, 36) = 0.08806	P=0.7684	**F (1, 36) = 85.39**	***** P<0.0001**	F (1, 36) = 2.521	P=0.1211	**0.0001**	**0.0001**	0.5941	0.8271
**MCP1/CCL2**	F (1, 35) = 1.611	P=0.2128	F (1, 35) = 1.262	P=0.2689	F (1, 35) = 0.9888	P=0.3269	0.9999	0.3554	0.9995	0.3828
***MCP3/CCL7**	F (1, 34) = 0.1284	P=0.7223	**F (1, 34) = 11.72**	**** P=0.0016**	F (1, 34) = 1.042	P=0.3145	0.1411	**0.045**	0.9851	0.7904
***MIP1A/CCL3**	F (1, 35) = 0.8145	P=0.3729	**F (1, 35) = 141.9**	***** P<0.0001**	F (1, 35) = 0.1139	P=0.7378	**0.0001**	**0.0001**	0.9914	0.8523
***MIP1B/CCL4**	F (1, 35) = 0.02258	P=0.8814	**F (1, 35) = 60.85**	***** P<0.0001**	F (1, 35) = 0.005541	P=0.9411	**0.0001**	**0.0001**	0.9999	0.9997
**MIP2**	F (1, 34) = 0.7632	P=0.3884	**F (1, 34) = 4.779**	*** P=0.0358**	F (1, 34) = 0.3940	P=0.5344	0.1422	0.8322	0.9996	0.7542
***RANTES/CCL5**	F (1, 35) = 0.02904	P=0.8657	F (1, 35) = 0.6350	P=0.4309	F (1, 35) = 0.1260	P=0.7248	0.9337	0.9874	0.9935	0.9999
**0**										
**BTC**	F (1, 35) = 0.01165	P=0.9147	F (1, 35) = 1.097	P=0.3020	F (1, 35) = 0.1368	P=0.7137	0.94	0.8911	0.9996	0.995
**LEPTIN**	F (1, 35) = 0.8437	P=0.3646	**F (1, 35) = 3.411**	**P=0.0732 (trend)**	F (1, 35) = 0.3758	P=0.5438	0.2036	0.9475	0.7495	0.9991
**VEGF**	F (1, 35) = 1.015	P=0.3207	F (1, 35) = 0.2390	P=0.6280	F (1, 35) = 0.08646	P=0.7705	0.9932	0.7649	0.8425	0.9774
**0**										
**IL2RA**	F (1, 35) = 0.1514	P=0.6996	F (1, 35) = 0.6647	P=0.4204	F (1, 35) = 0.4396	P=0.5116	0.9968	0.8759	0.9198	0.9994
**IL7RA**	F (1, 35) = 0.4950	P=0.4863	F (1, 35) = 1.144	P=0.2922	F (1, 35) = 1.027	P=0.3179	0.9982	0.6379	0.6647	0.9991
**ST2/IL33R**	F (1, 35) = 0.5490	P=0.4637	F (1, 35) = 1.592	P=0.2153	F (1, 35) = 0.4141	P=0.5241	0.502	0.9937	0.9999	0.7958

Supplementary Table S5. Luminex Cytokine Data - ANOVA Table for 5XFAD adrb2 cKO – female, brain homogenate.

Supplemental Table S5 - Luminex Data ANOVA Table for ADRB2 conditional KO - female data										
	FEMALE ADRB2 cKO (two-way ANOVA effects of SEX x TREATMENT)						Sidak's			
	INTERACTION		GENE		cKO		wt-con v 5x-con	wt-cKO v 5X-cKO	wt-con v wt-cKO	5X-con v 5X cKO
BAFF	F (1, 29) = 4.671	* P=0.0391	F (1, 29) = 82.74	*** P<0.0001	F (1, 29) = 3.890	P=0.0582 (trend)	*** 0.0001	*** 0.0001	0.9999	** 0.0074
GCSF	F (1, 28) = 2.401	P=0.1325	F (1, 28) = 5.450	* P=0.0270	F (1, 28) = 0.1282	P=0.7230	0.0354	0.9721	0.9203	0.3846
GMCSF	F (1, 29) = 5.429	* P=0.0270	F (1, 29) = 31.16	*** P<0.0001	F (1, 29) = 4.002	P=0.0549 (trend)	*** 0.0001	0.106	0.9993	0.0048
IFNA	F (1, 28) = 0.3197	P=0.5763	F (1, 28) = 2.661	P=0.1140	F (1, 28) = 1.500	P=0.2309	0.4492	0.9076	0.7102	0.9733
IFNG	F (1, 29) = 0.1945	P=0.6625	F (1, 29) = 3.468	P=0.0727 (trend)	F (1, 29) = 0.03950	P=0.8438	0.7953	0.376	0.9998	0.9744
IL1A	F (1, 29) = 0.3982	P=0.5329	F (1, 29) = 2.981	P=0.0949	F (1, 29) = 0.01639	P=0.8990	0.9076	0.3537	0.9964	0.953
IL1B	F (1, 29) = 0.8030	P=0.3776	F (1, 29) = 18.96	*** P=0.0002	F (1, 29) = 0.07143	P=0.7912	0.0038	0.0767	0.9915	0.8137
IL2	F (1, 28) = 4.338	* P=0.0465	F (1, 28) = 0.000	P>0.9999	F (1, 28) = 0.02311	P=0.8803	0.4828	0.4828	0.6525	0.2796
IL3	F (1, 29) = 0.01650	P=0.8987	F (1, 29) = 4.052	P=0.0535 (trend)	F (1, 29) = 0.4439	P=0.5105	0.4633	0.568	0.9953	0.9449
IL4	F (1, 29) = 0.1168	P=0.7350	F (1, 29) = 3.763	P=0.0622 (trend)	F (1, 29) = 0.05480	P=0.8165	0.4017	0.706	0.9939	0.9999
IL5	F (1, 28) = 0.8813	P=0.3559	F (1, 28) = 12.72	** P=0.0013	F (1, 28) = 0.1361	P=0.7150	0.0163	0.2474	0.9939	0.7547
IL6	F (1, 29) = 0.8877	P=0.3539	F (1, 29) = 2.211	P=0.1478	F (1, 29) = 0.3294	P=0.5704	0.9925	0.3255	0.8211	0.9968
IL7	F (1, 29) = 1.936	P=0.1747	F (1, 29) = 3.403	P=0.0753 (trend)	F (1, 29) = 0.0004796	P=0.9827	0.9963	0.1085	0.8549	0.7095
IL9	F (1, 29) = 0.6373	P=0.4312	F (1, 29) = 0.04168	P=0.8397	F (1, 29) = 0.4612	P=0.5024	0.9312	0.9888	0.9999	0.6498
IL10	F (1, 26) = 4.889	* P=0.0360	F (1, 26) = 4.474	* P=0.0442	F (1, 26) = 0.3295	P=0.5709	0.017	0.9999	0.2933	0.6077
IL12P70	F (1, 29) = 1.734	P=0.1982	F (1, 29) = 1.098	P=0.3033	F (1, 29) = 0.1261	P=0.7251	0.9995	0.3507	0.7641	0.8959
IL13	F (1, 29) = 0.5643	P=0.4586	F (1, 29) = 6.198	* P=0.0188	F (1, 29) = 0.03008	P=0.8635	0.6535	0.1077	0.9938	0.9084
IL15/IL15R	F (1, 29) = 0.06168	P=0.8056	F (1, 29) = 12.29	** P=0.0015	F (1, 29) = 1.696	P=0.2030	(trend) 0.0527	0.105	0.9442	0.6085
IL17A/CTLA8	F (1, 29) = 0.001709	P=0.9673	F (1, 29) = 5.881	* P=0.0218	F (1, 29) = 0.03566	P=0.8515	0.3275	0.3432	0.9999	0.9995
IL18	F (1, 29) = 0.3299	P=0.5702	F (1, 29) = 11.04	** P=0.0024	F (1, 29) = 0.3690	P=0.5483	0.0418	0.2182	0.9999	0.8051
IL19	F (1, 29) = 0.08797	P=0.7689	F (1, 29) = 7.787	** P=0.0092	F (1, 29) = 0.2855	P=0.5972	0.1465	0.3013	0.9998	0.9358
IL22	F (1, 29) = 1.036	P=0.3173	F (1, 29) = 6.069	* P=0.0199	F (1, 29) = 1.564	P=0.2211	0.0814	0.7745	0.9998	0.2528
IL23	F (1, 28) = 1.540	P=0.2249	F (1, 28) = 0.2818	P=0.5997	F (1, 28) = 0.3117	P=0.5811	0.9817	0.5966	0.9891	0.4412
IL25/IL17	F (1, 29) = 0.6038	P=0.4434	F (1, 29) = 1.105	P=0.3018	F (1, 29) = 0.09792	P=0.7566	0.9995	0.5954	0.9374	0.9923
IL27	F (1, 28) = 0.007137	P=0.9333	F (1, 28) = 1.439	P=0.2403	F (1, 28) = 3.309	P=0.0796 (trend)	0.9054	0.8349	0.7328	0.4337
IL28	F (1, 29) = 1.288	P=0.2657	F (1, 29) = 1.303	P=0.2631	F (1, 29) = 0.7999	P=0.3785	0.404	0.9999	0.9998	0.3538
IL31	F (1, 29) = 0.1529	P=0.6986	F (1, 29) = 7.306	* P=0.0114	F (1, 29) = 0.4419	P=0.5115	0.145	0.3723	0.9997	0.8605
IL33	F (1, 28) = 0.9206	P=0.3455	F (1, 28) = 2.529	P=0.1230	F (1, 28) = 0.3073	P=0.5838	0.3071	0.9855	0.8167	0.9957
*LIF	F (1, 28) = 7.745	** P=0.0095	F (1, 28) = 51.62	*** P<0.0001	F (1, 28) = 8.203	** P=0.0078	0.0001	0.0169	0.9999	0.0003
MCSF	F (1, 29) = 0.1536	P=0.6980	F (1, 29) = 25.63	*** P<0.0001	F (1, 29) = 0.2626	P=0.6122	0.0109	0.0021	0.9999	0.9148
SRANKL	F (1, 27) = 0.2009	P=0.6575	F (1, 27) = 2.667	P=0.1141	F (1, 27) = 0.01451	P=0.9050	0.4552	0.8889	0.9993	0.9825
TNFA	F (1, 29) = 0.6396	P=0.4304	F (1, 29) = 2.435	P=0.1295	F (1, 29) = 0.01558	P=0.9015	0.9739	0.3526	0.9647	0.969
0										
ENA78/LIX/CXCL5	F (1, 29) = 0.1117	P=0.7406	F (1, 29) = 10.41	** P=0.0031	F (1, 29) = 0.1889	P=0.6671	(trend) 0.0719	0.1791	0.9999	0.9508
EOTAXIN/CCL11	F (1, 29) = 0.009336	P=0.9237	F (1, 29) = 0.01283	P=0.9106	F (1, 29) = 1.965	P=0.1716	0.9998	0.9999	0.8867	0.6381
GROA/KC/CXCL1	F (1, 29) = 0.07382	P=0.7878	F (1, 29) = 7.079	* P=0.0126	F (1, 29) = 0.2228	P=0.6405	0.3576	0.1694	0.984	0.9997
*IP10/CXCL10	F (1, 30) = 2.039	P=0.1636	F (1, 30) = 42.39	*** P<0.0001	F (1, 30) = 0.2364	P=0.6303	0.0001	0.0039	0.9632	0.3964
MCP1/CCL2	F (1, 29) = 0.4394	P=0.5126	F (1, 29) = 9.717	** P=0.0041	F (1, 29) = 0.02297	P=0.8806	0.0506	0.3159	0.9961	0.94
*MCP3/CCL7	F (1, 28) = 2.654	P=0.1145	F (1, 28) = 6.834	* P=0.0142	F (1, 28) = 2.633	P=0.1159	0.0223	0.9333	0.9999	0.0508
*MIP1A/CCL3	F (1, 29) = 5.568	* P=0.0252	F (1, 29) = 74.21	*** P<0.0001	F (1, 29) = 5.884	* P=0.0217	0.0001	0.0005	0.9999	0.0018
*MIP1B/CCL4	F (1, 27) = 3.676	P=0.0659 (trend)	F (1, 27) = 84.81	***P<0.0001	F (1, 27) = 3.440	P=0.0746 (trend)	0.0001	0.0001	0.9999	0.0212
MIP2	F (1, 29) = 1.684	P=0.2046	F (1, 29) = 7.347	* P=0.0112	F (1, 29) = 0.06257	P=0.8042	0.0347	0.7886	0.81	0.8638
*RANTES/CCL5	F (1, 29) = 0.4064	P=0.5288	F (1, 29) = 19.37	*** P=0.0001	F (1, 29) = 0.04632	P=0.8311	0.052	0.0047	0.9736	0.9946
0										
BTC	F (1, 29) = 2.878	P=0.1005	F (1, 29) = 0.4141	P=0.5250	F (1, 29) = 0.001093	P=0.9738	0.9189	0.3608	0.7417	0.5449
LEPTIN	F (1, 29) = 1.180	P=0.2863	F (1, 29) = 13.34	** P=0.0010	F (1, 29) = 0.1065	P=0.7465	0.2912	0.0083	0.8551	0.9528
VEGF	F (1, 29) = 2.555	P=0.1208	F (1, 29) = 1.088	P=0.3056	F (1, 29) = 0.6038	P=0.4434	0.2657	0.9913	0.977	0.2149
0										
IL2RA	F (1, 28) = 0.2541	P=0.6181	F (1, 28) = 0.08050	P=0.7787	F (1, 28) = 0.1039	P=0.7496	0.9714	0.9998	0.9999	0.9408
IL7RA	F (1, 29) = 0.0002233	P=0.9882	F (1, 29) = 3.484	P=0.0721 (trend)	F (1, 29) = 0.9794	P=0.3305	0.5853	0.5841	0.9527	0.8914
ST2/IL33R	F (1, 28) = 0.9916	P=0.3279	F (1, 28) = 0.8278	P=0.3707	F (1, 28) = 0.09308	P=0.7625	0.5834	0.9999	0.9874	0.7581

Supplementary Table S6. Luminex Cytokine Data - ANOVA Table for 5XFAD adrb1 cKO – male, brain homogenate.

Supplemental Table S6 - Luminex Data ANOVA Table for ADRB1 conditional KO - male data										
	MALE ADRB1 cKO (two-way ANOVA effects of SEX x TREATMENT)						Sidak's			
	INTERACTION		GENE		cKO		wt-con v 5x-con	wt-cKO v 5X-cKO	wt-con v wt-cKO	5X-con v 5X cKO
BAFF	F (1, 44) = 3.660	P=0.0623 (trend)	F (1, 44) = 54.38	*** P<0.0001	F (1, 44) = 2.102	P=0.1542	*** 0.0001	** 0.0015	0.9958	(trend) 0.0845
GCSF	F (1, 44) = 1.806	P=0.1859	F (1, 44) = 0.000	P>0.9999	F (1, 44) = 0.5769	P=0.4516	0.8184	0.8184	0.9897	0.4633
GMCSF	F (1, 44) = 1.576	P=0.2160	F (1, 44) = 14.74	*** P=0.0004	F (1, 44) = 0.4416	P=0.5098	** 0.0032	0.2661	0.5513	0.9893
IFNA	F (1, 44) = 1.499	P=0.2274	F (1, 44) = 0.4307	P=0.5151	F (1, 44) = 0.002549	P=0.9600	0.5705	0.9908	0.8797	0.8448
IFNG	F (1, 44) = 1.887	P=0.1765	F (1, 44) = 0.006756	P=0.9349	F (1, 44) = 0.6189	P=0.4357	0.7718	0.8385	0.4369	0.9895
IL1A	F (1, 44) = 0.08166	P=0.7764	F (1, 44) = 2.470	P=0.1232	F (1, 44) = 0.7349	P=0.3959	0.5818	0.8406	0.8894	0.9905
IL1B	F (1, 44) = 4.463	* P=0.0404	F (1, 44) = 4.851	* P=0.0329	F (1, 44) = 0.2444	P=0.6235	* 0.0153	0.9999	0.2584	0.6981
IL2	F (1, 44) = 0.3884	P=0.5364	F (1, 44) = 1.536	P=0.2218	F (1, 44) = 0.3366	P=0.5647	0.5794	0.9874	0.8699	0.9999
IL3	F (1, 44) = 1.709	P=0.1979	F (1, 44) = 2.088	P=0.1555	F (1, 44) = 0.004735	P=0.9455	0.2126	0.9999	0.8054	0.8578
IL4	F (1, 44) = 1.136	P=0.2923	F (1, 44) = 0.07361	P=0.7874	F (1, 44) = 0.1818	P=0.6719	0.821	0.968	0.7559	0.9856
IL5	F (1, 43) = 0.1429	P=0.7073	F (1, 43) = 1.167	P=0.2861	F (1, 43) = 0.2362	P=0.6294	0.7779	0.9788	0.9999	0.9552
IL6	F (1, 44) = 0.04279	P=0.8371	F (1, 44) = 2.541	P=0.1181	F (1, 44) = 0.0001481	P=0.9903	0.8009	0.6096	0.9999	0.9998
IL7	F (1, 44) = 0.2521	P=0.6181	F (1, 44) = 0.1537	P=0.6969	F (1, 44) = 0.3580	P=0.5527	0.9999	0.9514	0.9999	0.9021
IL9	F (1, 44) = 0.2405	P=0.6263	F (1, 44) = 0.1627	P=0.6886	F (1, 44) = 0.1564	P=0.6944	0.9999	0.9515	0.9529	0.9999
IL10	F (1, 40) = 0.07494	P=0.7857	F (1, 40) = 4.375	* P=0.0429	F (1, 40) = 0.01109	P=0.9167	0.3503	0.6026	0.9981	0.9999
IL12P70	F (1, 44) = 0.03521	P=0.8520	F (1, 44) = 0.1263	P=0.7240	F (1, 44) = 0.4881	P=0.4884	0.9922	0.9999	0.9938	0.9529
IL13	F (1, 44) = 0.05049	P=0.8232	F (1, 44) = 6.369	* P=0.0153	F (1, 44) = 2.584	P=0.1151	0.2139	0.3759	0.8027	0.5942
IL15/IL15R	F (1, 44) = 0.3379	P=0.5640	F (1, 44) = 1.621	P=0.2096	F (1, 44) = 0.3379	P=0.5640	0.5833	0.9807	0.8833	0.9999
IL17A/CTLA8	F (1, 44) = 0.6108	P=0.4387	F (1, 44) = 0.02357	P=0.8787	F (1, 44) = 0.5266	P=0.4719	0.9865	0.9433	0.9999	0.7492
IL18	F (1, 44) = 0.3146	P=0.5777	F (1, 44) = 7.776	** P=0.0078	F (1, 44) = 0.04340	P=0.8359	0.0863 (trend)	0.4068	0.9715	0.9985
IL19	F (1, 44) = 0.2405	P=0.6263	F (1, 44) = 0.1627	P=0.6886	F (1, 44) = 0.1564	P=0.6944	0.9999	0.9515	0.9529	0.9999
IL22	F (1, 41) = 7.695	** P=0.0083	F (1, 41) = 8.504	** P=0.0057	F (1, 41) = 0.02806	P=0.8678	** 0.0011	0.9999	0.188	0.2301
IL23	F (1, 40) = 0.002888	P=0.9574	F (1, 40) = 0.1673	P=0.6847	F (1, 40) = 2.488	P=0.1226	0.9954	0.9986	0.6913	0.7441
IL25/IL17	F (1, 43) = 0.7836	P=0.3810	F (1, 43) = 0.1491	P=0.7013	F (1, 43) = 0.8893	P=0.3509	0.8408	0.9946	0.9999	0.6063
IL27	F (1, 43) = 0.1517	P=0.6988	F (1, 43) = 0.3645	P=0.5492	F (1, 43) = 1.006	P=0.3215	0.9329	0.9998	0.9881	0.7923
IL28	F (1, 44) = 0.7632	P=0.3871	F (1, 44) = 0.04064	P=0.8412	F (1, 44) = 0.05017	P=0.8238	0.9093	0.9826	0.9029	0.9847
IL31	F (1, 43) = 2.276	P=0.1387	F (1, 43) = 2.633	P=0.1120	F (1, 43) = 6.718e-006	P=0.9979	0.1293	0.9999	0.7573	0.74
IL33	F (1, 44) = 1.229	P=0.2736	F (1, 44) = 0.1994	P=0.6574	F (1, 44) = 1.174	P=0.2844	0.9836	0.7274	0.9999	0.4225
*LIF	F (1, 44) = 4.577	* P=0.0380	F (1, 44) = 10.20	** P=0.0026	F (1, 44) = 0.1163	P=0.7347	** 0.0019	0.9149	0.6108	0.3033
MCSF	F (1, 43) = 1.219	P=0.2758	F (1, 43) = 15.19	*** P=0.0003	F (1, 43) = 0.5667	P=0.4557	** 0.0035	0.2099	0.5925	0.9985
SRANKL	F (1, 44) = 1.553	P=0.2193	F (1, 44) = 0.3985	P=0.5312	F (1, 44) = 0.06294	P=0.8031	0.572	0.9875	0.9298	0.7537
TNFA	F (1, 44) = 0.2687	P=0.6068	F (1, 44) = 0.01770	P=0.8948	F (1, 44) = 0.01770	P=0.8948	0.9845	0.9979	0.9845	0.9979
ENA78/LIX/CXCL5	F (1, 43) = 1.156	P=0.2883	F (1, 43) = 8.065	** P=0.0069	F (1, 43) = 1.246	P=0.2705	* 0.0302	0.6371	0.9999	0.4341
EOTAXIN/CCL11	F (1, 44) = 0.2621	P=0.6112	F (1, 44) = 3.992	(trend) P=0.0519	F (1, 44) = 0.1140	P=0.7373	0.2924	0.7587	0.9999	0.9594
GROA/KC/CXCL1	F (1, 43) = 1.114	P=0.2970	F (1, 43) = 4.172	* P=0.0473	F (1, 43) = 1.046	P=0.3121	0.1223	0.9343	0.9999	0.4861
*IP10/CXCL10	F (1, 43) = 5.582	* P=0.0227	F (1, 43) = 61.77	*** P<0.0001	F (1, 43) = 0.02774	P=0.8685	*** 0.0001	** 0.0012	0.432	0.2758
MCP1/CCL2	F (1, 44) = 0.05688	P=0.8126	F (1, 44) = 14.20	*** P=0.0005	F (1, 44) = 0.1926	P=0.6629	* 0.0274	(trend) 0.0639	0.9821	0.9998
*MCP3/CCL7	F (1, 44) = 1.927	P=0.1720	F (1, 44) = 32.39	*** P<0.0001	F (1, 44) = 0.2489	P=0.6203	*** 0.0001	* 0.0157	0.9523	0.5672
*MIP1A/CCL3	F (1, 44) = 1.904	P=0.1746	F (1, 44) = 53.68	*** P<0.0001	F (1, 44) = 1.448	P=0.2353	*** 0.0001	*** 0.0005	0.9999	0.2665
*MIP1B/CCL4	F (1, 43) = 0.2825	P=0.5978	F (1, 43) = 40.37	*** P<0.0001	F (1, 43) = 0.2313	P=0.6330	*** 0.0001	*** 0.0008	0.9999	0.9229
MIP2	F (1, 44) = 0.2072	P=0.6512	F (1, 44) = 6.520	* P=0.0142	F (1, 44) = 0.3171	P=0.5762	0.1472	0.4656	0.9999	0.9242
*RANTES/CCL5	F (1, 43) = 6.186	* P=0.0168	F (1, 43) = 17.78	*** P=0.0001	F (1, 43) = 0.3642	P=0.5493	*** 0.0001	0.635	0.1374	0.5589
BTC	F (1, 44) = 0.04188	P=0.8388	F (1, 44) = 0.6918	P=0.4100	F (1, 44) = 0.3530	P=0.5555	0.9866	0.9196	0.9674	0.9978
LEPTIN	F (1, 44) = 2.499	P=0.1210	F (1, 44) = 16.91	*** P=0.0002	F (1, 44) = 0.000	P>0.9999	*** 0.0009	0.2848	0.7155	0.7155
VEGF	F (1, 44) = 0.4383	P=0.5114	F (1, 44) = 0.4972	P=0.4845	F (1, 44) = 1.111	P=0.2976	0.8091	0.9999	0.9978	0.6511
IL2RA	F (1, 43) = 3.044	(trend) P=0.0882	F (1, 43) = 0.7180	P=0.4015	F (1, 43) = 0.004328	P=0.9479	0.2737	0.9489	0.6149	0.6601
IL7RA	F (1, 44) = 1.547	P=0.2202	F (1, 44) = 2.119	P=0.1525	F (1, 44) = 0.01571	P=0.9008	0.2286	0.9998	0.8969	0.8083
ST2/IL33R	F (1, 44) = 1.547	P=0.2201	F (1, 44) = 2.362	P=0.1315	F (1, 44) = 0.007735	P=0.9303	0.9993	0.2045	0.8231	0.8854

Supplementary Table S7. Luminex Cytokine Data - ANOVA Table for 5XFAD adrb1 cKO – female, brain homogenate.

**Supplemental Table S7 - Luminex Data ANOVA Table for ADRB1 conditional KO - female data**										
	FEMALE ADRB1 cKO (two-way ANOVA effects of SEX x TREATMENT)						Sidak's			
	**INTERACTION**		**GENE**		**cKO**		**wt-con v 5x-con**	**wt-cKO v 5X-cKO**	**wt-con v wt-cKO**	**5X-con v 5X cKO**
**BAFF**	F (1, 44) = 0.005189	P=0.9429	**F (1, 44) = 71.51**	***** P<0.0001**	F (1, 44) = 0.01525	P=0.9023	***** 0.0001**	***** 0.0001**	0.9999	0.9999
**GCSF**	F (1, 44) = 0.9164	P=0.3437	F (1, 44) = 0.1539	P=0.6967	F (1, 44) = 0.01995	P=0.8883	0.9909	0.8161	0.9648	0.9027
**GMCSF**	F (1, 44) = 0.03757	P=0.8472	**F (1, 44) = 7.282**	**** P=0.0098**	F (1, 44) = 0.7738	P=0.3838	0.1746	0.2944	0.9097	0.9813
**IFNA**	F (1, 44) = 2.144	P=0.1503	F (1, 44) = 0.3546	P=0.5546	**F (1, 44) = 5.442**	*** P=0.0243**	0.4839	0.9561	*** 0.0402**	0.9561
**IFNG**	F (1, 44) = 0.7616	P=0.3876	**F (1, 44) = 10.97**	**** P=0.0019**	F (1, 44) = 0.01729	P=0.8960	0.3192	*** 0.0197**	0.9277	0.9751
**IL1A**	F (1, 44) = 0.03599	P=0.8504	**F (1, 44) = 4.131**	*** P=0.0482**	F (1, 44) = 0.3239	P=0.5722	0.4091	0.589	0.9729	0.998
**IL1B**	F (1, 44) = 0.06511	P=0.7998	**F (1, 44) = 14.06**	***** P=0.0005**	F (1, 44) = 1.341	P=0.2531	*** 0.0276**	**(trend) 0.0679**	0.7901	0.9497
**IL2**	F (1, 44) = 0.1104	P=0.7413	F (1, 44) = 0.6257	P=0.4332	F (1, 44) = 0.08109	P=0.7772	0.9959	0.8954	0.9999	0.9874
**IL3**	F (1, 44) = 0.02300	P=0.8802	F (1, 44) = 0.2981	P=0.5878	F (1, 44) = 0.1113	P=0.7402	0.9977	0.9801	0.9999	0.9949
**IL4**	F (1, 44) = 0.1964	P=0.6598	F (1, 44) = 0.09411	P=0.7605	F (1, 44) = 0.02353	P=0.8788	0.9999	0.974	0.9889	0.9993
**IL5**	**F (1, 44) = 5.969**	*** P=0.0186**	**F (1, 44) = 6.728**	*** P=0.0128**	F (1, 44) = 0.003639	P=0.9522	0.9999	**** 0.0036**	0.2948	0.3413
**IL6**	F (1, 44) = 0.4153	P=0.5227	F (1, 44) = 1.831	P=0.1829	F (1, 44) = 0.2658	P=0.6088	0.5135	0.9789	0.8841	0.9999
**IL7**	F (1, 44) = 0.6542	P=0.4230	F (1, 44) = 1.109	P=0.2980	F (1, 44) = 0.1520	P=0.6985	0.9997	0.5796	0.8715	0.9971
**IL9**	F (1, 44) = 0.0001954	P=0.9889	F (1, 44) = 0.9303	P=0.3401	F (1, 44) = 0.01468	P=0.9041	0.9337	0.94	0.9999	0.9999
**IL10**	F (1, 38) = 0.03555	P=0.8514	F (1, 38) = 0.4044	P=0.5286	F (1, 38) = 0.4055	P=0.5281	0.9959	0.9668	0.9598	0.9966
**IL12P70**	F (1, 44) = 1.206	P=0.2782	**F (1, 44) = 6.015**	*** P=0.0182**	F (1, 44) = 0.4474	P=0.5071	0.8141	**(trend) 0.0617**	0.9968	0.6263
**IL13**	F (1, 44) = 1.924	P=0.1724	**F (1, 44) = 4.177**	*** P=0.0470**	F (1, 44) = 0.008152	P=0.9285	0.9841	**(trend) 0.0755**	0.8365	0.7625
**IL15/IL15R**	F (1, 44) = 1.303	P=0.2599	F (1, 44) = 0.06730	P=0.7965	F (1, 44) = 0.0006730	P=0.9794	0.7952	0.9537	0.8977	0.8817
**IL17A/CTLA8**	F (1, 44) = 0.1125	P=0.7389	F (1, 44) = 1.669	P=0.2032	F (1, 44) = 0.2287	P=0.6349	0.6938	0.9387	0.9652	0.9999
**IL18**	F (1, 43) = 1.829	P=0.1833	**F (1, 43) = 21.47**	***** P<0.0001**	F (1, 43) = 0.1852	P=0.6691	**(trend) 0.0913**	***** 0.0006**	0.9479	0.6089
**IL19**	F (1, 44) = 0.0001954	P=0.9889	F (1, 44) = 0.9303	P=0.3401	F (1, 44) = 0.01468	P=0.9041	0.9337	0.94	0.9999	0.9999
**IL22**	F (1, 42) = 0.07733	P=0.7823	F (1, 42) = 0.008865	P=0.9254	F (1, 42) = 0.3037	P=0.5845	0.9999	0.9982	0.9994	0.9655
**IL23**	F (1, 42) = 0.8745	P=0.3551	**F (1, 42) = 4.226**	*** P=0.0461**	F (1, 42) = 1.298	P=0.2610	0.8964	0.1521	0.9998	0.4983
**IL25/IL17**	F (1, 44) = 1.952	P=0.1694	F (1, 44) = 0.1593	P=0.6917	F (1, 44) = 1.683	P=0.2013	0.9292	0.6119	0.2301	0.9999
**IL27**	F (1, 44) = 0.2664	P=0.6083	F (1, 44) = 0.7400	P=0.3943	F (1, 44) = 0.007400	P=0.9318	0.9987	0.8053	0.9968	0.9885
**IL28**	F (1, 44) = 1.274	P=0.2652	F (1, 44) = 1.714	P=0.1973	F (1, 44) = 0.000	P>0.9999	0.9999	0.3195	0.8938	0.8938
**IL31**	F (1, 42) = 0.3677	P=0.5475	**F (1, 42) = 6.337**	*** P=0.0157**	F (1, 42) = 0.3677	P=0.5475	0.5563	0.1245	0.9999	0.8575
**IL33**	F (1, 44) = 0.4474	P=0.5071	F (1, 44) = 1.582	P=0.2150	F (1, 44) = 0.2707	P=0.6055	0.5478	0.9894	0.8746	0.9999
***LIF**	F (1, 43) = 0.2421	P=0.6252	**F (1, 43) = 26.96**	***** P<0.0001**	F (1, 43) = 3.799e-005	P=0.9951	**** 0.0081**	***** 0.0008**	0.9947	0.9946
**MCSF**	F (1, 44) = 2.227	P=0.1427	**F (1, 44) = 19.10**	***** P<0.0001**	F (1, 44) = 1.010	P=0.3205	0.1784	***** 0.0006**	0.9948	0.2971
**SRANKL**	**F (1, 44) = 6.543**	*** P=0.0141**	F (1, 44) = 0.02434	P=0.8767	F (1, 44) = 0.03161	P=0.8597	0.2242	0.3336	0.3423	0.2176
**TNFA**	F (1, 44) = 0.3913	P=0.5348	**F (1, 44) = 4.248**	*** P=0.0452**	F (1, 44) = 0.2692	P=0.6064	0.7807	0.2326	0.9999	0.8889
**ENA78/LIX/CXCL5**	F (1, 44) = 0.003151	P=0.9555	**F (1, 44) = 10.01**	**** P=0.0028**	F (1, 44) = 0.1147	P=0.7364	0.1265	0.1062	0.9977	0.9994
**EOTAXIN/CCL11**	F (1, 44) = 0.1636	P=0.6878	F (1, 44) = 2.128	P=0.1517	F (1, 44) = 0.1058	P=0.7465	0.9149	0.579	0.9765	0.9999
**GROA/KC/CXCL1**	F (1, 44) = 2.468	P=0.1233	**F (1, 44) = 12.88**	***** P=0.0008**	F (1, 44) = 0.1483	P=0.7021	0.5038	**** 0.0028**	0.5336	0.8757
***IP10/CXCL10**	F (1, 44) = 0.04237	P=0.8379	**F (1, 44) = 52.53**	***** P<0.0001**	F (1, 44) = 1.361	P=0.2497	***** 0.0001**	***** 0.0001**	0.9377	0.8069
**MCP1/CCL2**	F (1, 41) = 1.723	P=0.1966	**F (1, 41) = 25.12**	***** P<0.0001**	F (1, 41) = 0.1726	P=0.6800	*** 0.0448**	***** 0.0003**	0.9446	0.673
***MCP3/CCL7**	F (1, 42) = 1.045	P=0.3125	**F (1, 42) = 18.58**	***** P<0.0001**	F (1, 42) = 0.6411	P=0.4278	**(trend) 0.0962**	**** 0.002**	0.9997	0.6182
***MIP1A/CCL3**	F (1, 44) = 0.6427	P=0.4270	**F (1, 44) = 68.14**	***** P<0.0001**	F (1, 44) = 0.5072	P=0.4801	***** 0.0001**	***** 0.0001**	0.9999	0.7462
***MIP1B/CCL4**	F (1, 44) = 0.1638	P=0.6876	**F (1, 44) = 50.95**	***** P<0.0001**	F (1, 44) = 0.2056	P=0.6525	***** 0.0001**	***** 0.0001**	0.9999	0.9579
**MIP2**	F (1, 44) = 1.295	P=0.2613	F (1, 44) = 2.633	P=0.1118	F (1, 44) = 0.03669	P=0.8490	0.995	0.2103	0.9408	0.824
***RANTES/CCL5**	F (1, 44) = 2.501	P=0.1209	**F (1, 44) = 26.28**	***** P<0.0001**	F (1, 44) = 0.8082	P=0.3736	**(trend) 0.0624**	***** 0.0001**	0.9816	0.3033
**BTC**	F (1, 44) = 0.006773	P=0.9348	F (1, 44) = 0.6242	P=0.4337	F (1, 44) = 0.1475	P=0.7028	0.979	0.9554	0.9992	0.9956
**LEPTIN**	F (1, 44) = 1.598	P=0.2128	**F (1, 44) = 23.00**	***** P<0.0001**	F (1, 44) = 0.9682	P=0.3305	0.0637	***** 0.0004**	0.9994	0.3978
**VEGF**	F (1, 43) = 0.3698	P=0.5463	F (1, 43) = 1.012	P=0.3202	F (1, 43) = 0.002630	P=0.9593	0.7086	0.9976	0.9831	0.9918
**IL2RA**	F (1, 43) = 1.145	P=0.2905	F (1, 43) = 1.739	P=0.1943	F (1, 43) = 1.483	P=0.2299	0.3286	0.9996	0.3917	0.9999
**IL7RA**	F (1, 44) = 0.1555	P=0.6952	F (1, 44) = 0.5692	P=0.4546	F (1, 44) = 0.04968	P=0.8246	0.9984	0.8876	0.9999	0.9874
**ST2/IL33R**	F (1, 43) = 0.8580	P=0.3595	**F (1, 43) = 6.832**	*** P=0.0123**	F (1, 43) = 3.951	P=0.0532	**(trend) 0.059**	0.6741	0.1777	0.9098

Supplementary Table S8. Luminex Cytokine Data - ANOVA Table for Sex effects in 5XFAD mice (*from ADRB2 cKO study*).

Supplemental Table S8 - Luminex Data ANOVA Table for ADRB2 conditional KO - sex comparison								
	ADRB2 cKO (two-way ANOVA effects of SEX x TREATMENT)						Sidak's	
	INTERACTION		SEX		TREATMENT		sex effect in 5XFAD-veh	sex effect in 5XFAD-cKO
BAFF	F (3, 64) = 11.01	***P<0.0001	F (1, 64) = 10.72	**P=0.0017	F (3, 64) = 54.55	***P<0.0001	***	
GCSF	F (3, 62) = 0.9999	P=0.3990	F (1, 62) = 1.591	P=0.2119	F (3, 62) = 4.415	P=0.0070	ns	
GMCSF	F (3, 64) = 3.562	P=0.0189	F (1, 64) = 2.624	P=0.1102	F (3, 64) = 13.12	P<0.0001	**	
IFNA	F (3, 63) = 1.264	P=0.2944	F (1, 63) = 0.0009	P=0.9763	F (3, 63) = 0.5366	P=0.6589		
IFNG	F (3, 63) = 0.9085	P=0.4421	F (1, 63) = 0.1293	P=0.7204	F (3, 63) = 1.170	P=0.3284		
IL1A	F (3, 63) = 1.160	P=0.3323	F (1, 63) = 0.1297	P=0.7199	F (3, 63) = 0.7548	P=0.5237		
IL1B	F (3, 64) = 2.131	P=0.1050	F (1, 64) = 6.152	*P=0.0158	F (3, 64) = 10.37	***P<0.0001	*	
IL2	F (3, 63) = 1.051	P=0.3761	F (1, 63) = 0.2635	P=0.6095	F (3, 63) = 1.104	P=0.3541		
IL3	F (3, 65) = 0.7155	P=0.5463	F (1, 65) = 0.03758	P=0.8469	F (3, 65) = 3.923	P=0.0123	ns	
IL4	F (3, 63) = 0.1935	P=0.9005	F (1, 63) = 0.01852	P=0.8922	F (3, 63) = 2.827	P=0.0456	ns	
IL5	F (3, 63) = 1.156	P=0.3337	F (1, 63) = 0.3731	P=0.5435	F (3, 63) = 3.465	P=0.0213	ns	
IL6	F (3, 62) = 1.152	P=0.3353	F (1, 62) = 1.359	P=0.2482	F (3, 62) = 0.7532	P=0.5247		
IL7	F (3, 64) = 1.427	P=0.2430	F (1, 64) = 0.8291	P=0.3659	F (3, 64) = 0.8820	P=0.4552		
IL9	F (3, 63) = 0.5862	P=0.6263	F (1, 63) = 0.9666	P=0.3293	F (3, 63) = 0.9330	P=0.4301		
IL10	F (3, 60) = 3.257	P=0.0277	F (1, 60) = 1.282	P=0.2621	F (3, 60) = 0.9035	P=0.4448	**	
IL12P70	F (3, 64) = 1.457	P=0.2347	F (1, 64) = 0.2119	P=0.6469	F (3, 64) = 0.3968	P=0.7557		
IL13	F (3, 64) = 2.182	P=0.0987	F (1, 64) = 0.2353	P=0.6292	F (3, 64) = 1.134	P=0.3422		
IL15/IL15R	F (3, 64) = 2.587	P=0.0607	F (1, 64) = 1.859	P=0.1775	F (3, 64) = 3.655	P=0.0170	*	
IL17A/CTLA8	F (3, 63) = 0.7595	P=0.5210	F (1, 63) = 0.05389	P=0.8172	F (3, 63) = 3.096	P=0.0331	ns	
IL18	F (3, 64) = 2.366	P=0.0792	F (1, 64) = 0.5645	P=0.4552	F (3, 64) = 4.468	P=0.0065	ns	
IL19	F (3, 64) = 4.247	P=0.0085	F (1, 64) = 1.310	P=0.2567	F (3, 64) = 0.8350	P=0.4796	0.06	*
IL22	F (3, 63) = 1.412	P=0.2477	F (1, 63) = 2.520	P=0.1174	F (3, 63) = 4.558	P=0.0059	ns	
IL23	F (3, 61) = 2.327	P=0.0834	F (1, 61) = 0.0060	P=0.9384	F (3, 61) = 0.2044	P=0.8929		
IL25/IL17	F (3, 64) = 1.046	P=0.3785	F (1, 64) = 0.3751	P=0.5424	F (3, 64) = 0.07090	P=0.9753		
IL27	F (3, 63) = 1.357	P=0.2641	F (1, 63) = 4.060	P=0.0482	F (3, 63) = 1.774	P=0.1612	ns	0.09
IL28	F (3, 64) = 1.335	P=0.2708	F (1, 64) = 0.8418	P=0.3623	F (3, 64) = 1.410	P=0.2481		
IL31	F (3, 63) = 0.8091	P=0.4935	F (1, 63) = 0.7806	P=0.3803	F (3, 63) = 3.517	P=0.0201	ns	
IL33	F (3, 62) = 1.283	P=0.2881	F (1, 62) = 0.4542	P=0.5028	F (3, 62) = 0.8066	P=0.4950		
*LIF	F (3, 62) = 17.18	P<0.0001	F (1, 62) = 20.02	P<0.0001	F (3, 62) = 33.51	P<0.0001	***	
MCSF	F (3, 63) = 2.655	P=0.0561	F (1, 63) = 4.831	P=0.0316	F (3, 63) = 11.46	P<0.0001	0.08	*
SRANKL	F (3, 61) = 1.372	P=0.2599	F (1, 61) = 1.979	P=0.1646	F (3, 61) = 0.4386	P=0.7262		
TNFA	F (3, 63) = 0.3495	P=0.7896	F (1, 63) = 1.120e-005	P=0.9973	F (3, 63) = 1.457	P=0.2347		
ENA78/LIX/CXCL5	F (3, 64) = 1.718	P=0.1722	F (1, 64) = 0.5326	P=0.4682	F (3, 64) = 4.033	P=0.0109	ns	
EOTAXIN/CCL11	F (3, 64) = 1.604	P=0.1972	F (1, 64) = 1.521	P=0.2220	F (3, 64) = 0.1485	P=0.9303		
GROA/KC/CXCL1	F (3, 64) = 1.002	P=0.3977	F (1, 64) = 1.141	P=0.2895	F (3, 64) = 2.328	P=0.0828		
*IP10/CXCL10	F (3, 66) = 4.609	P=0.0055	F (1, 66) = 20.49	P<0.0001	F (3, 66) = 33.82	P<0.0001	***	*
MCP1/CCL2	F (3, 64) = 1.913	P=0.1363	F (1, 64) = 0.1709	P=0.6807	F (3, 64) = 3.515	P=0.0200	ns	
*MCP3/CCL7	F (3, 62) = 4.727	P=0.0049	F (1, 62) = 9.003	P=0.0039	F (3, 62) = 6.013	P=0.0012	***	
*MIP1A/CCL3	F (3, 64) = 10.92	P<0.0001	F (1, 64) = 24.77	P<0.0001	F (3, 64) = 61.54	P<0.0001	***	*
*MIP1B/CCL4	F (3, 62) = 7.298	P=0.0003	F (1, 62) = 13.94	P=0.0004	F (3, 62) = 54.66	P<0.0001	***	
MIP2	F (3, 63) = 0.5462	P=0.6525	F (1, 63) = 1.311	P=0.2566	F (3, 63) = 5.062	P=0.0033	ns	
*RANTES/CCL5	F (3, 64) = 3.102	P=0.0327	F (1, 64) = 2.161	P=0.1464	F (3, 64) = 5.358	P=0.0024	0.09	*
BTC	F (3, 64) = 0.6711	P=0.5729	F (1, 64) = 0.2376	P=0.6276	F (3, 64) = 1.294	P=0.2842		
LEPTIN	F (3, 64) = 1.958	P=0.1292	F (1, 64) = 0.6033	P=0.4402	F (3, 64) = 5.735	P=0.0015	ns	
VEGF	F (3, 64) = 0.9159	P=0.4383	F (1, 64) = 11.03	P=0.0015	F (3, 64) = 1.561	P=0.2076	**	
IL2RA	F (3, 63) = 0.3257	P=0.8067	F (1, 63) = 5.922	P=0.0178	F (3, 63) = 0.1580	P=0.9242	ns	
IL7RA	F (3, 64) = 1.742	P=0.1673	F (1, 64) = 0.3278	P=0.5690	F (3, 64) = 1.054	P=0.3749		
ST2/IL33R	F (3, 63) = 1.493	P=0.2250	F (1, 63) = 0.05901	P=0.8089	F (3, 63) = 0.05731	P=0.9818		

Supplementary Table S9. Definition of region abbreviations used in describing clusters from brain-wide analyses.

Region	Abbrev.	Subregion
Cortical subplate	EP	Endopiriform nucleus
	CLA	Claustrum
	CTXsp	Cortical subplate
Olfactory	PIR	Piriform area
	PAA	Piriform-amygdalar area
	TT	Taenia tecta
	TR	Postpiriform transition area
	AON	Anterior olfactory nucleus
	OLF	Olfactory
Isocortex	PL	Prelimbic
	AI	Agranular insula
	ORB	Orbital
	MO	Motor
	SS	Somatosensory
	ACA	Anterior cingulate
	RSP	Retrosplenial
	GU	Gustatory
Retrohippocampal	ENT	Entorhinal
Cortical plate	CTXpl	Cortical plate
Striatum	CP	Caudoputamen
	LSX	Lateral septal complex
	STR	Striatum
Cerebellum	UVU	Uvula (IX)
	PYR	Pyramus (VIII)
	FL	Flocculus
	PFL	Paraflocculus
	COPY	Copula pyramidis
	PRM	Paramedian lobule
	AN	Ansiform lobule
Midbrain	IC	Inferior colliculus
	SC	Superior colliculus
	PAG	Periaqueductal gray
	MB	Midbrain
Thalamus	TH	Thalamus
Hypothalamus	PM	Premammillary nucleus
	MM	Medial mammillary nucleus
	LHA	Lateral hypothalamic area
	PVa	Periventricular hypothalamic nucleus, anterior part
	ARH	Arcuate hypothalamic nucleus
	PVi	Periventricular hypothalamic nucleus, intermediate part
	DMH	Dorsomedial nucleus of the hypothalamus
	PV	Periventricular hypothalamic nucleus
	HY	Hypothalamus
Pons	PG	Pontine gray
	V	Motor nucleus of trigeminal
	TRN	Tegmental reticular nucleus
	PRNc	Pontine reticular nucleus, caudal part
	PSV	Principal sensory nucleus of the trigeminal
	RPO	Nucleus raphe pontis
	PRNr	Pontine reticular nucleus
	P	Pons
Medulla	SPVI	Spinal nucleus of the trigeminal, interpolar part
	SPVC	Spinal nucleus of the trigeminal, caudal part
	PARN	Parvicellular reticular nucleus
	MDRN	Medullary reticular nucleus
	IRN	Intermediate reticular nucleus
	GRN	Gigantocellular reticular nucleus
	MY	Medulla
fiber tracts	fx	columns of the fornix
	cst	corticospinal tract
	sptV	spinal tract of the trigeminal nerve
	och	optic chiasm
	mlf	medial longitudinal fascicle
	mcp	middle cerebellar peduncle
	icp	inferior cerebellar peduncle
	arb	arbor vitae

Supplementary Table S10. Information on valid clusters includes the contrast, effect direction, label, FDR q value, and adjusted p-value threshold as well as the cluster ID, volume (in mm^3), general region, and top 4 regions (with the percentage of total cluster volume indicated).

5XFAD > NC (Aβ aggregates; q < 0.2; p < 0.025; label density)							
**Cluster**	**Volume**	**CoG**	**~Region**	**Top Region 1**	**Top Region 2**	**Top Region 3**	**Top Region 4**
**15****	0.0077	275,161,81.8	Olfactory	PIR (71%)	AIv2/3 (29%)		
**8***	0.0281	296,247,27.7		PIR (96%)	PAA (4%)		
**5****	0.0531	228,130,68.9		AON (96%)	TTv (3%)	PIR (1%)	
**2*******	6.9842	239,208,174	Isocortex	Isocortex (88%)	LSX (5%)	OLF (3%)	STR (1%)
**1*******	11.6101	273,318,114	Cortical plate	CTXpl (49%)	TH (17%)	CTXsp (11%)	MBmot (8%)
**6***	0.0386	196,314,59.7	Hypothalamus	Mmme (88%)	PMd (6%)	LHA (2%)	fx (1%)
**9***	0.0159	254,377,88.1	Pons, sensory related	PSV (68%)	P (24%)	mcp (7%)	
**4*******	0.1003	228,385,86.5	Pons	P (51%)	V (24%)	PRNr (12%)	PRNc (7%)
**3******	0.1703	237,451,70.1	Medulla, sensory related	SPVI (37%)	PARN (35%)	SPVC (16%)	MDRNd (11%)
**12******	0.0116	232,404,75.6	Medulla, motor related	PARN (100%)			
**7*******	0.0384	205,457,61.6		IRN (62%)	MDRNv (27%)	GRN (11%)	

5XFAD > NC (Reactive microglia; q < 0.2; p < 0.025; label density)							
**Cluster**	**Volume**	**CoG**	**~Region**	**Top Region 1**	**Top Region 2**	**Top Region 3**	**Top Region 4**
**4*******	0.0174	273,168,80.9	Olfactory	PIR (42%)	EPd (22%)	AIv5 (21%)	OLF (10%)
**6******	0.0135	274,169,97.6	Agranular insula	AIv5 (52%)	AIv6a (17%)	CLA (12%)	AId5 (12%)
**5****	0.0159	294,184,101		AId6a (61%)	AId5 (28%)	GU5 (4%)	CLA (4%)
**1****	23.2712	267,273,147	Cortical plate	CTXpl (75%)	TH (8%)	CTXsp (6%)	MBmot (3%)
**3****	0.1545	228,381,183	Midbrain, sensory related	ICc (48%)	ICd (31%)	ICe (14%)	MB (5%)
**7***	0.0098	213,349,67.4	Pons, motor related	TRN (60%)	PG (39%)	cst (1%)	
**2****	1.6336	222,422,71.3	Medulla, motor related	MY-mot (55%)	MY-sen (12%)	PRNr (11%)	P-mot (8%)

5XFAD < NC (TH fibers; q < 0.4; p < 0.011; label density)							
**Cluster**	**Volume**	**CoG**	**~Region**	**Top Region 1**	**Top Region 2**	**Top Region 3**	**Top Region 4**
**8***	0.0719	72,289,203	Somatosensory	SSp-bfd5 (34%)	SSp-bfd4 (26%)	SSp-bfd2/3 (12%)	SSs5 (9%)
**2****	0.9355	166,186,189	Anterior cingulate	ACA (39%)	MO (22%)	PL (15%)	ORB (11%)
**35***	0.0082	152,248,240	Retrosplenial	RSPagl1 (60%)	MOp1 (24%)	SSp-tr1 (11%)	RSPagl2/3 (4%)
**1***	1.8833	60.3,235,114	Isocortex	Isocortex (68%)	EP (8%)	CP (5%)	CLA (4%)
**34****	0.0086	46.8,343,75.6	Hippocampal	ENTl3 (48%)	ENTl5 (48%)	TR (4%)	
**17***	0.0304	182,481,126	Cerebellar cortex	UVU (96%)	arb (3%)		
**68***	0.002	150,487,160		PYR (75%)	COPY (25%)		
**37***	0.0078	181,476,160		PYR (76%)	arb (24%)		
**36***	0.0081	93.3,398,99.1		FL (85%)	PFL (15%)		
**39****	0.0071	82.1,387,105		FL (89%)	PFL (11%)		
**21******	0.0219	80.3,471,118		PRM (100%)			
**63****	0.0024	91.5,431,207		ANcr1 (100%)			
**6***	0.0748	174,330,204	Midbrain, sensory related	SCsg (52%)	SCzo (21%)	RSPv1 (19%)	RSPv2/3 (5%)
**5****	0.1199	176,246,30.7	Hypothalamus	HY (64%)	och (20%)	fiber tracts (16%)	
**52***	0.0041	181,398,85.7	Pons	P (35%)	RPO (29%)	mlf (16%)	PRNc (16%)
**47***	0.0051	106,428,50.8	fiber tracts	icp (71%)	sptV (25%)	MY (4%)	

5XFAD < NC (TH+ DA cells; q < 0.4; p < 0.011; cell density)							
**Cluster**	**Volume**	**CoG**	**~Region**	**Top Region 1**	**Top Region 2**	**Top Region 3**	**Top Region 4**
**22***	0.0034	168,323,147	Midbrain, motor related	PAG (100%)			
**5******	0.0101	176,246,30.7	Hypothalamus	ARH (48%)	PVpo (29%)	PVa (11%)	PVi (8%)
**62***	0.0018	181,267,60.5		PVi (95%)	DMH (5%)		
**46***	0.0051	181,280,59.3		PVi (68%)	DMH (32%)		
