# Supplementary material for: Impact of noradrenergic inhibition on neuroinflammation and pathophysiology in mouse models of Alzheimer’s disease
Source: J Neuroinflammation. 2024 Dec 18;21:322. doi: 10.1186/s12974-024-03306-1 (PMC11657531; doi:10.1186/s12974-024-03306-1)
Supplement: Supplementary file 2 — Supplementary Material 2 [file 12974_2024_3306_MOESM2_ESM.pdf]

Supplemental Figures

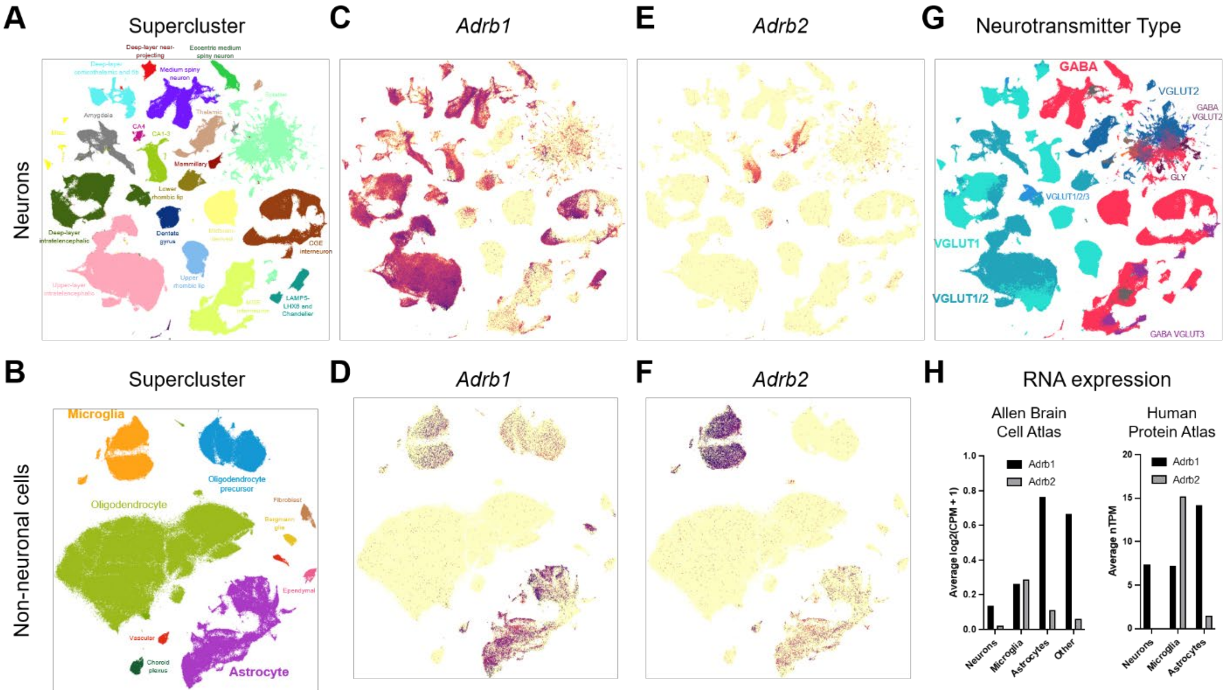

Supplemental Figure S1. Expression of *Adrb1* and *Adrb2* across neuronal and non-neuronal cell types in the adult human brain, visualized using single-cell RNA-sequencing data from the Allen Brain Cell Atlas (portal.brain-map.org/atlases-and-data/bkp/abc-atlas) (Siletti et al., 2023). **A-B)** tSNE plots showing superclusters of major cell types for **(A)** neurons and **(B)** non-neuronal cells. **C-F)** tSNE plots indicating the expression of *Adrb1* (**C-D**) and *Adrb2* (**E-F**) across neuronal (**C, E**) and non-neuronal **(D, F)** cell types. Expression intensity is color-coded, with darker colors representing higher expression levels. **G)** Neurotransmitter type classification of neuronal clusters, highlighting cell types expressing markers for neurotransmitters such as GABA and glutamate (e.g., VGLUT1, VGLUT2). The data were retrieved from the Allen Brain Cell Atlas(1) (Siletti et al. 2023). **H)** Using this data and data from the Human Protein Atlas (www.proteinatlas.org)(2) (Karlsson et al., 2021), we quantified the average expression of *Adrb1* and *Adrb2* across broad cell-type categories. CPM = counts per million. nTPM = normalized transcripts per million.

1. Siletti K, Hodge R, Mossi Albiach A, Lee KW, Ding SL, Hu L, et al. Transcriptomic diversity of cell types across the adult human brain. Science. 2023;382(6667):eadd7046.
2. Karlsson M, Zhang C, Mear L, Zhong W, Digre A, Katona B, et al. A single-cell type transcriptomics map of human tissues. Sci Adv. 2021;7(31).

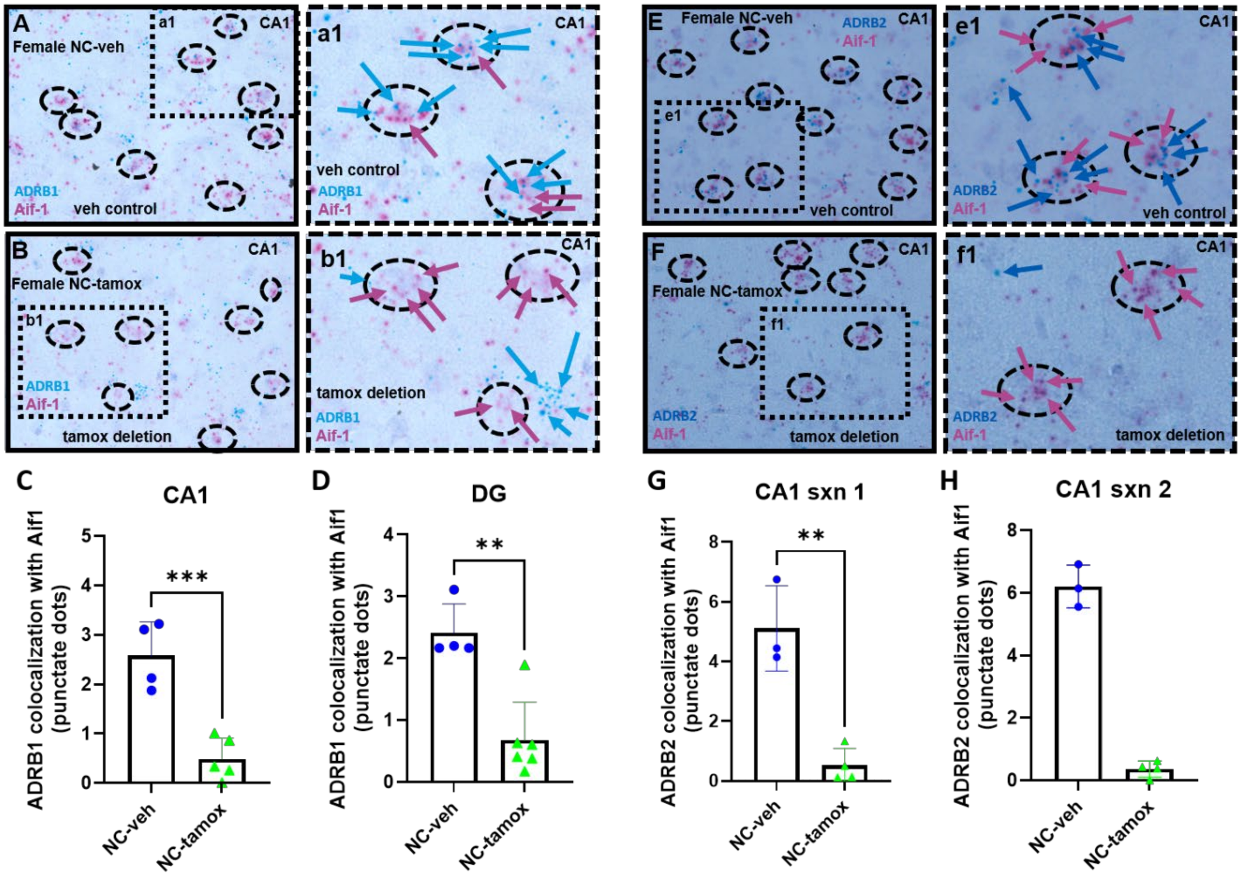

Supplemental Figure S2. RNAScope quantification of colocalization of adrb1 and adrb2 expression with allograft inflammatory factor 1 (aif1; also known as ionized calcium binding adapter molecule 1; iba1) expression in microglia. **A and B)** Representative images of RNAscope dual labelling of aif1 (red) and adrb1 (green). Dashed ovals indicate area around aif1 clusters as approximation of microglial cell bodies for quantification of adrb1 puncta. **A1 and B1**) Higher magnification representation of the dashed rectangular areas in **A** and **B** with colored arrows indicating representative punctate label. Green arrows indicate adrb1 puncta. Red arrows indicate aif1 puncta. **C and D)** Quantification of average number of adrb1 puncta within each aif1-positive cluster. **E and F)** Representative images of RNAscope dual labelling of aif1 (red) and adrb2 (green). Dashed ovals indicate area around aif1 clusters as approximation of microglial cell bodies for quantification of adrb1 puncta. **E1 and F1**) Higher magnification representation of the dashed rectangular areas in **E** and **F** with colored arrows indicating representative punctate label. Green arrows indicate adrb2 puncta. Red arrows indicate aif1 puncta. **G and H)** Quantification of average number of adrb1 puncta within each aif1-positive cluster.

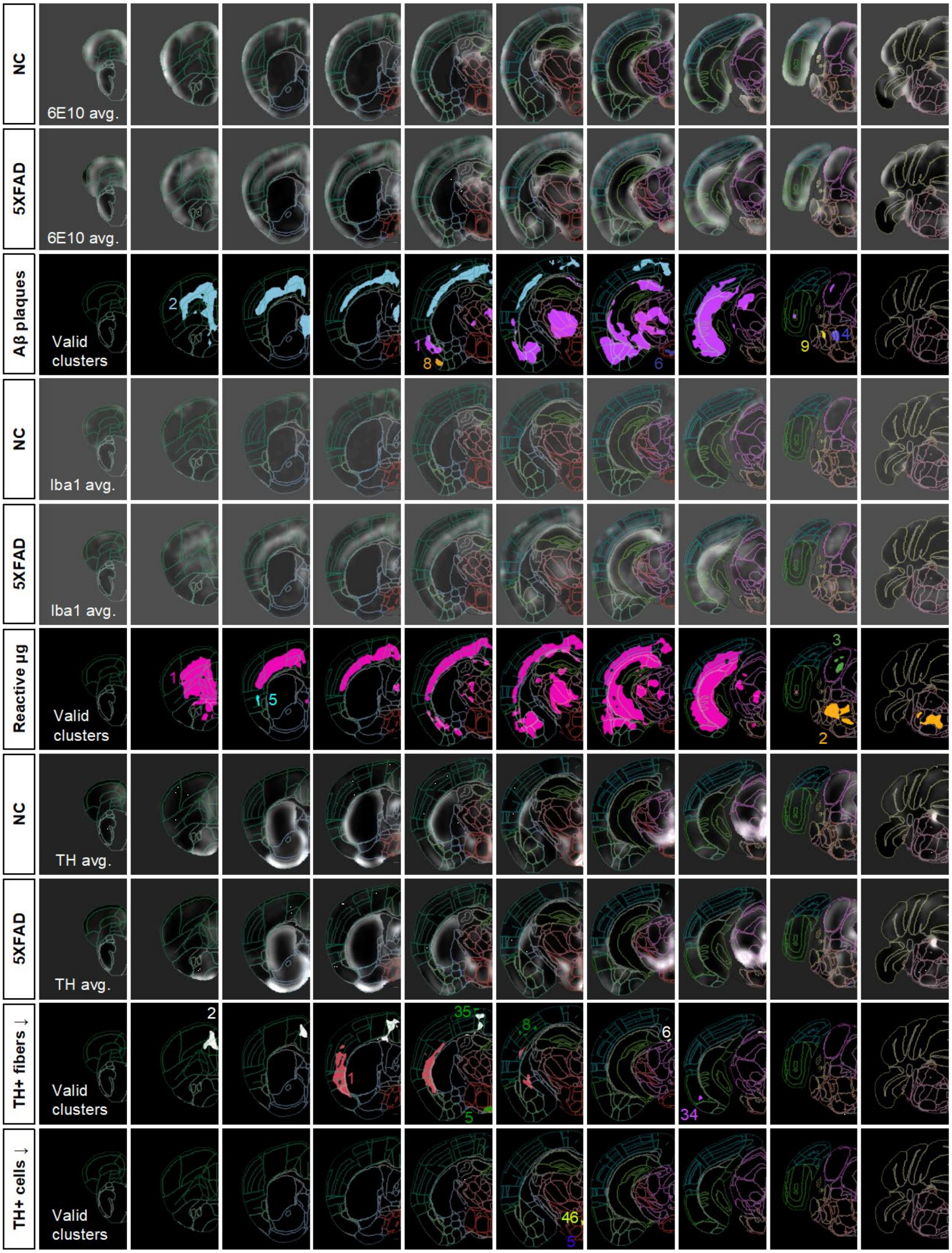

NC
6E10 avg.
5XFAD
6E10 avg.
Aβ plaques
Valid clusters
NC
Iba1 avg.
5XFAD
Iba1 avg.
Reactive µg
Valid clusters
NC
TH avg.
5XFAD
TH avg.
TH+ fibers ↓
Valid clusters
TH+ cells ↓
Valid clusters

Supplemental Figure S3. **Coronal slices showing averaged immunofluorescence and valid clusters.** Brains from aged 5XFAD (6.5 months) and non-carrier (NC) control mice were immunostained, cleared, imaged in 3D with LSFM and analyzed with UNRAVEL. Immunofluorescence labeling was preprocessed to minimize autofluorescence and non-specific staining. This involved rolling ball background subtraction (pixel radius of 20) to remove autofluorescence. Non-specific staining from the mouse 6E10 antibody was zeroed out with an Ilastik-based segmentation of capillaries and the brain's surfaces. Likewise, signal from reactive and aggregated microglia was isolated by segmenting them and zeroing out other voxels. No mask was used for TH labeling. Autofluorescence images were registered with an iDISCO+/LSFM-specific average template brain aligned to the CCFv3 Allen brain atlas. Transformation matrices from this were used to warp the preprocessed immunofluorescence images to atlas space for voxel-wise statistics. Each brain was z-scored and smoothed. Images for each immunolabel and group were averaged (avg.) together. Images were visualized with FSLeyes. The display range was set to -0.3 – 1 for 6E10, -0.3 – 0.8 for reactive microglia, and -0.3 – 2 for TH. Valid clusters were randomly colored and their IDs are shown. Detailed info on each cluster is available in **Supplementary Table S10**. The images were overlaid with a semi-transparent wireframe version of the Allen brain atlas.

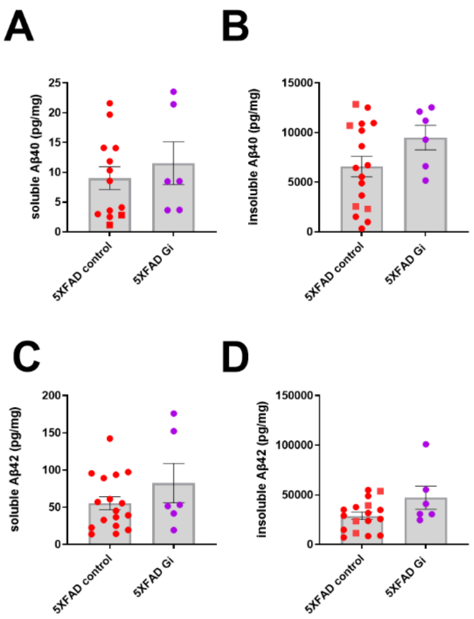

Supplemental Figure S4. No effects of chemogenetic inhibition of locus coeruleus noradrenergic neurons were detected on cortical tissue concentrations of amyloid beta. Bar graphs depict brain concentrations of soluble and insoluble **A,B)** Aβ40 and **C,D)** Aβ42 in female 5XFAD control mice and 5XFAD mice with chemogenetic inhibition (Gi) of the locus coeruleus.

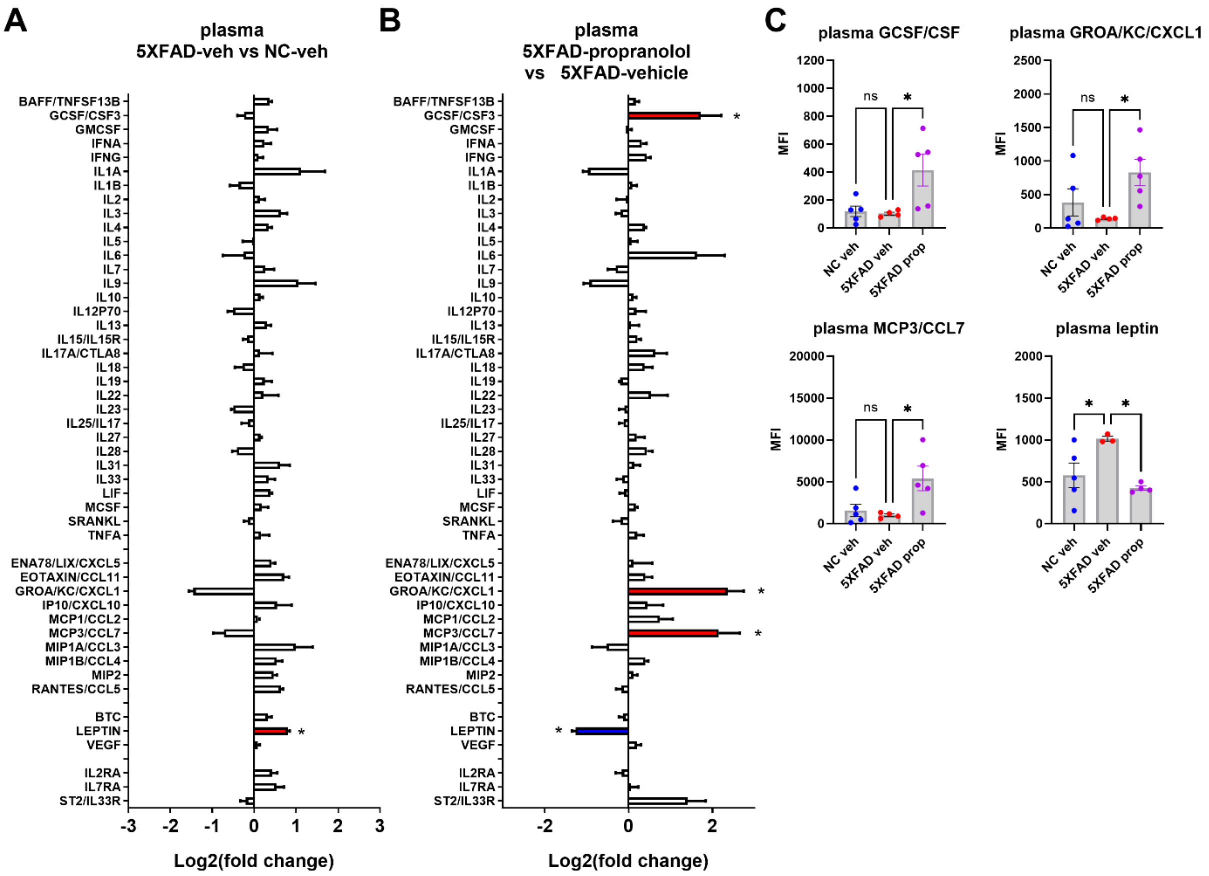

Supplemental Figure S5. Pharmacological inhibition of beta-adrenergic receptors with propranolol potentiates systemic inflammation and attenuates leptin in the 5XFAD mouse model of amyloidosis. Log2-fold change graphs depict effects of **A)** 5XFAD (5XFAD-veh/non-carrier (NC)-veh); n=5) in vehicle-treated mice and **B)** propranolol (5XFAD-prop/5XFAD-veh; n=6) in 5XFAD mice. **C)** Bar graphs indicate raw data from proteins affected by propranolol, as indicated in panel **B**. *$p < 0.05$; Sidak's post-hoc comparison of means following one-way ANOVA.

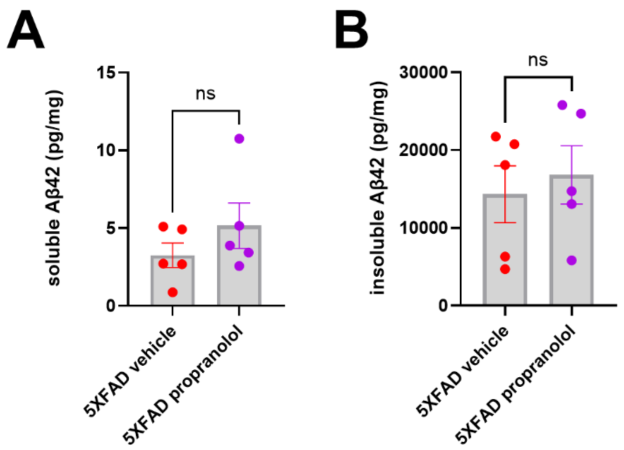

Supplemental Figure S6. No effects of propranolol were detected on frontal cortex concentrations of amyloid beta in 5XFAD mice. Bar graphs depict brain concentrations of **A)** soluble and **B)** insoluble Aβ42.

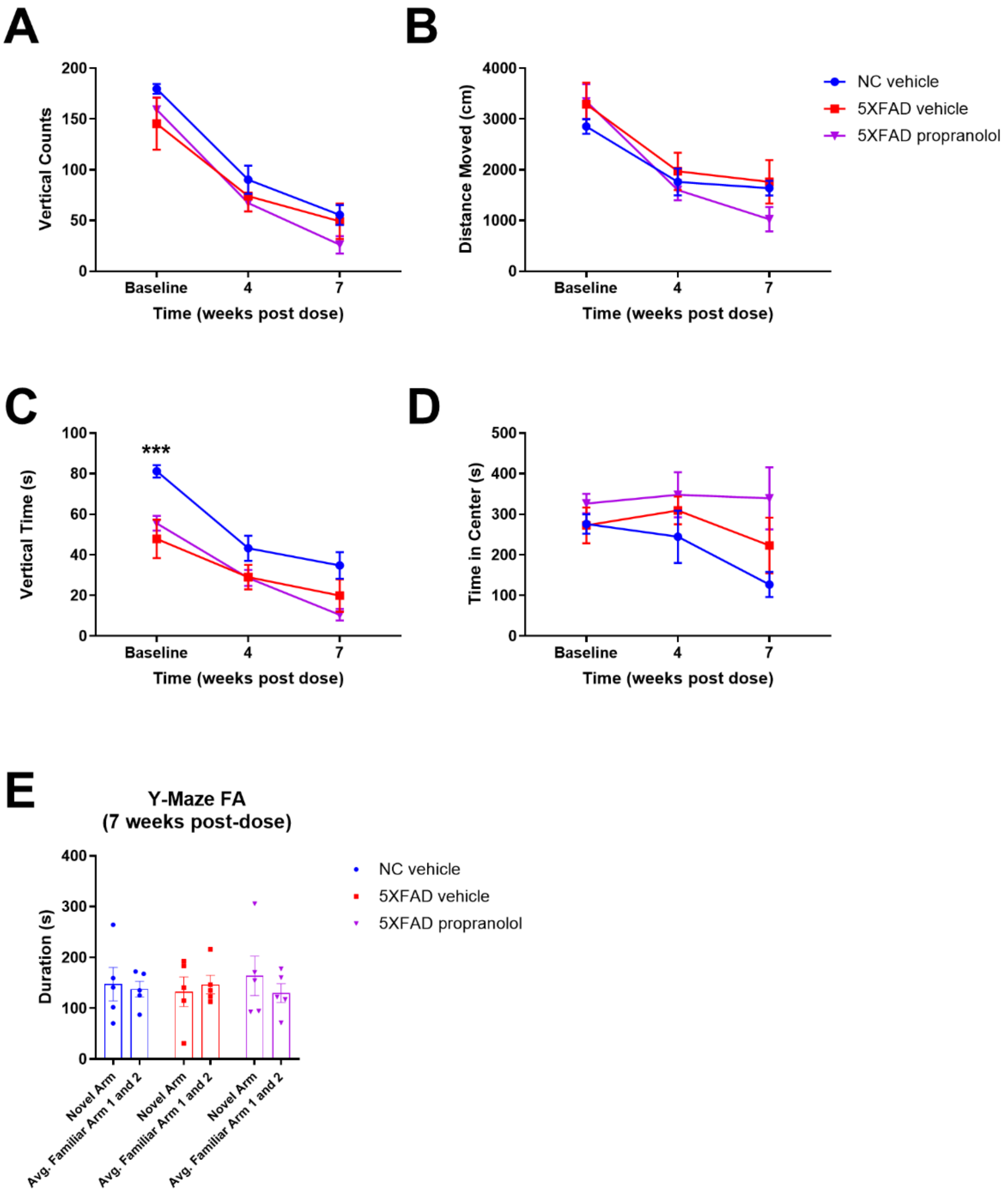

Supplemental Figure S7. Propranolol did not affect behavior in the activity chamber **(A-D)** or Y-maze **(E)** in 5XFAD mice. **A-D)** Line graphs depict Vertical Counts, Distance Moved, Vertical Time, and Time in the Center for the Activity Chamber assay. At baseline, 5XFAD mice showed less vertical time than non-carrier controls. **E)** Bar graphs depict duration in novel versus familiar arms after 7 weeks of dosing with propranolol. ***p < 0.001 for non-carrier-vehicle vs 5XFAD-vehicle Dunnett's post-hoc following two-way ANOVA.

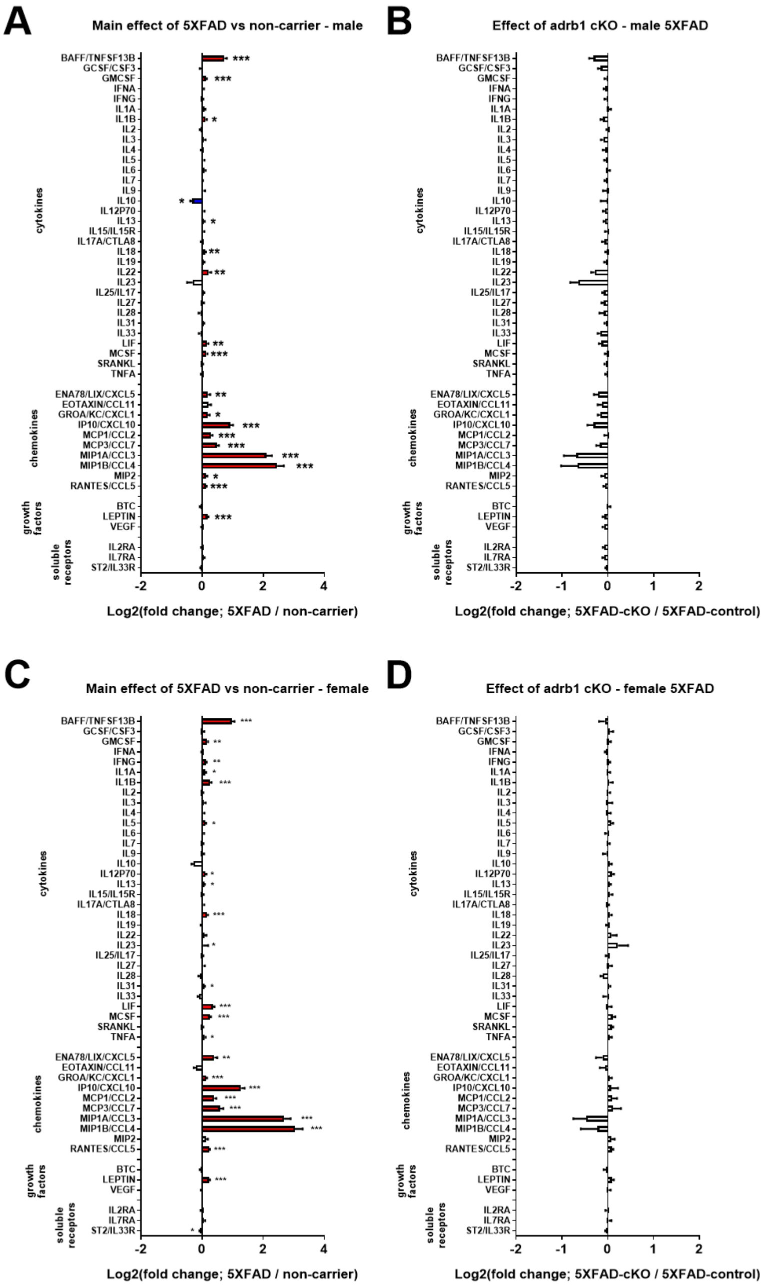

Supplemental Figure S8. Effects of conditional knockout of adrb1 in microglia in 5XFAD and non-carrier mice. Log2-fold graphs show fold changes across a panel of immune-related markers in response to **A)** 5XFAD in male mice and **B)** adrb1 cKO in male 5XFAD mice. Likewise, log2-fold graphs show immune regulation from **H)** 5XFAD in female mice and **I)** adrb1 cKO in female 5XFAD mice. For **A,C)** * indicates the main effects. For **B,D)** * indicates post-hoc Sidak's comparison of means following two-way ANOVA (5XFAD x adrb1 cKO). *p < 0.05, **p < 0.01, ***p < 0.001, ****p < 0.0001.

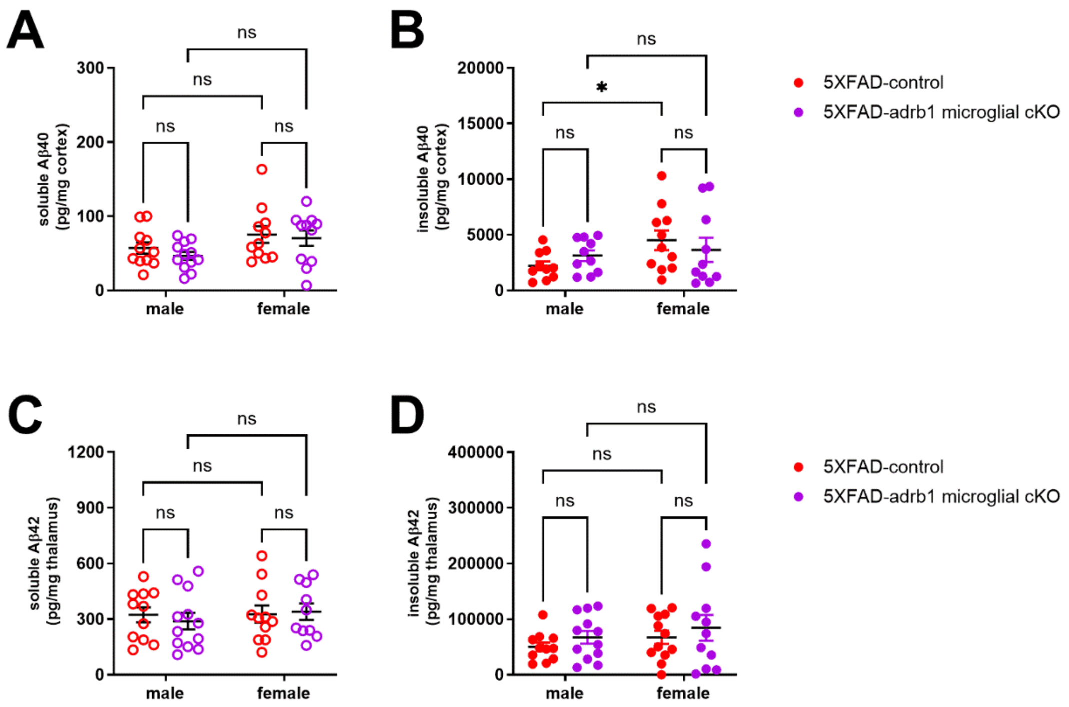

Supplemental Figure S9. No effects of conditional KO of adrb1 were detected on brain tissue concentrations of amyloid beta in 5XFAD mice. Bar graphs depict brain concentrations of soluble and insoluble **A,B)** Aβ40 (cortex) and **C,D)** Aβ42 (thalamus). *$p < 0.05$; Sidak's post-hoc comparison of means following two-way ANOVA (sex x adrb1 cKO).

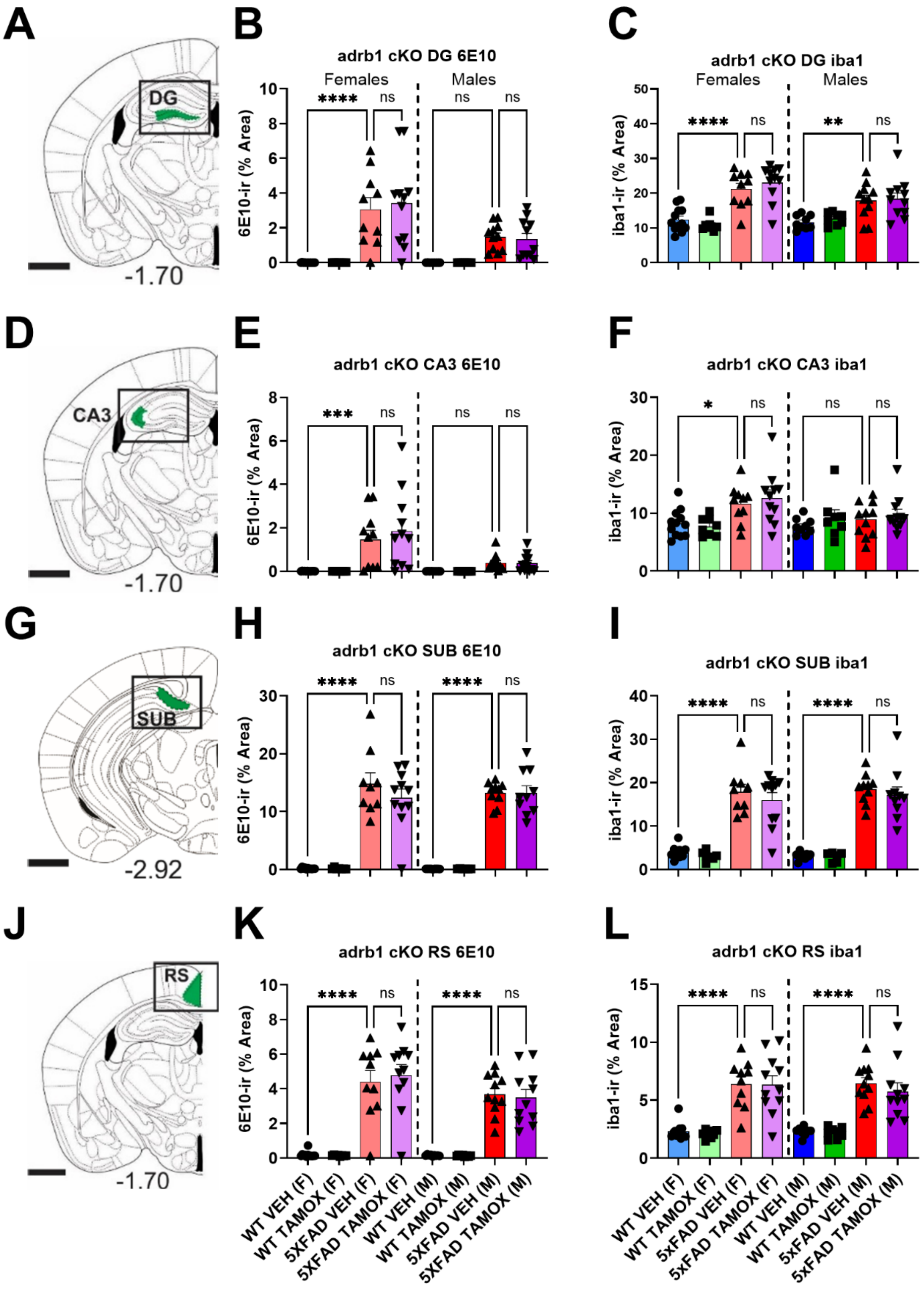
A
DG
-1.70
B
adrb1 cKO DG 6E10
Females
Males
6E10-ir (% Area)
C
adrb1 cKO DG iba1
iba1-ir (% Area)
D
CA3
E
adrb1 cKO CA3 6E10
F
adrb1 cKO CA3 iba1
G
SUB
-2.92
H
adrb1 cKO SUB 6E10
I
adrb1 cKO SUB iba1
J
RS
K
adrb1 cKO RS 6E10
L
adrb1 cKO RS iba1
WT VEH (F)
WT TAMOX (F)
5XFAD VEH (F)
5XFAD TAMOX (F)
WT VEH (M)
WT TAMOX (M)
5XFAD VEH (M)
5XFAD TAMOX (M)
5xFAD VEH (F)
5xFAD TAMOX (F)
5xFAD VEH (M)
5xFAD TAMOX (M)

Supplemental Figure S10. Conditional knockdown of adrb1 in mouse microglia did not increase 6E10 or iba1 immunoreactivity (ir) in 5XFAD mice. Bar graphs depict 6E10-ir or iba1-ir in the dentate gyrus (DG), CA3 region of the hippocampus, subiculum (SUB), or retrosplenial cortex (RS). *p < .05, **p < 0.01, ***p < 0.001, ****p < 0.0001; Sidak's post-hoc comparison of means following two-way ANOVA (5XFAD x adrb1 cKO) for each sex.

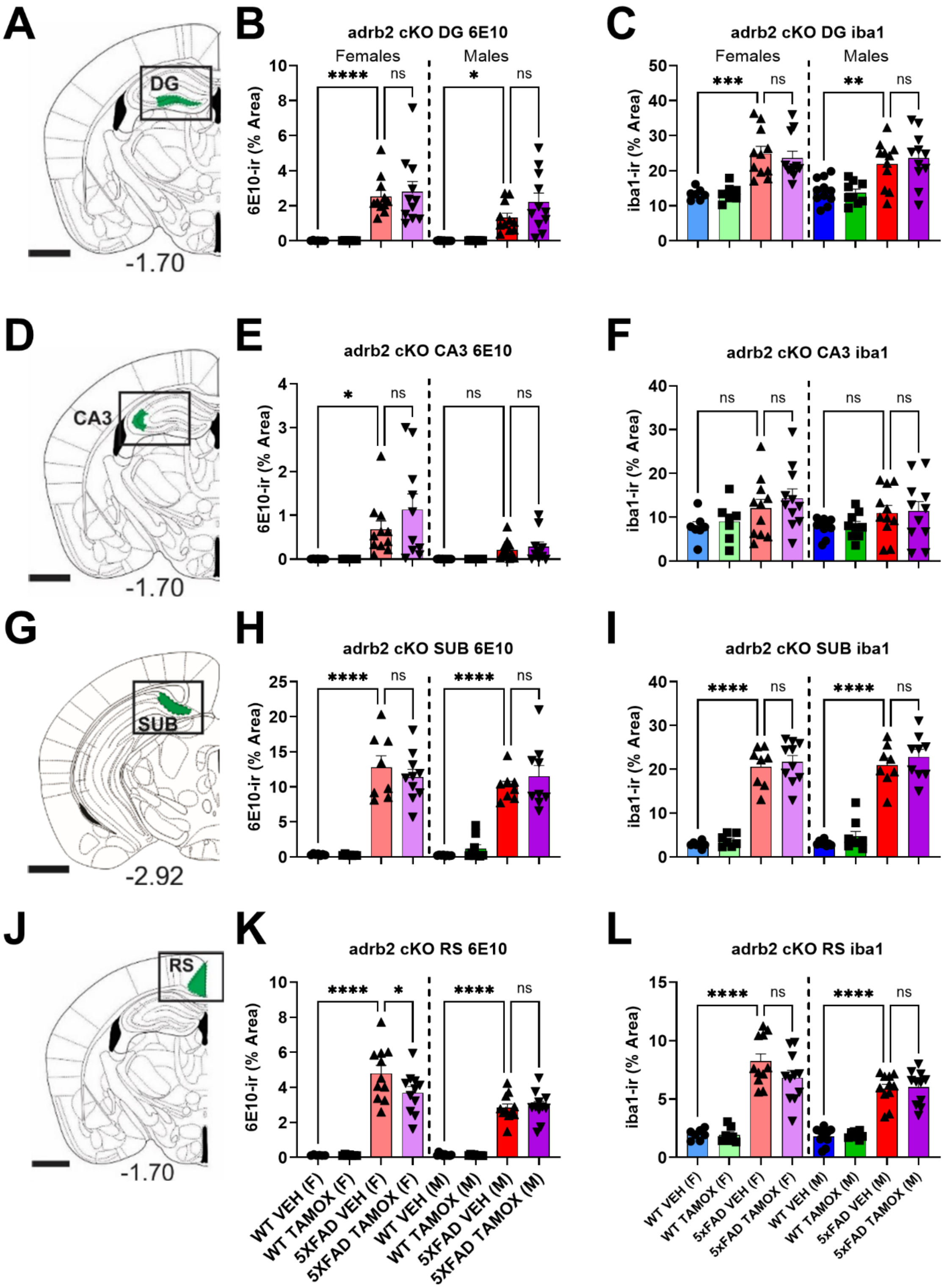
A
DG
-1.70
B
adrb2 cKO DG 6E10
Females
Males
6E10-ir (% Area)
C
adrb2 cKO DG iba1
iba1-ir (% Area)
D
CA3
-1.70
E
adrb2 cKO CA3 6E10
F
adrb2 cKO CA3 iba1
G
SUB
-2.92
H
adrb2 cKO SUB 6E10
I
adrb2 cKO SUB iba1
J
RS
-1.70
K
adrb2 cKO RS 6E10
L
adrb2 cKO RS iba1
WT VEH (F)
WT TAMOX (F)
5XFAD VEH (F)
5XFAD TAMOX (F)
WT VEH (M)
WT TAMOX (M)
5XFAD VEH (M)
5XFAD TAMOX (M)
5xFAD VEH (F)
5xFAD TAMOX (F)
5xFAD VEH (M)
5xFAD TAMOX (M)

Supplemental Figure S11. Conditional knockdown of adrb2 in mouse microglia did not increase 6E10 or iba1 immunoreactivity (ir) in 5XFAD mice. Bar graphs depict 6E10-ir or iba1-ir in the dentate gyrus (DG), CA3 region of the hippocampus, subiculum (SUB), or retrosplenial cortex (RS). *$p < 0.05$, **$p < 0.01$, ***$p < 0.001$, ****$p < 0.0001$; Sidak's post-hoc comparison of means following two-way ANOVA (5XFAD x adrb2 cKO) for each sex.

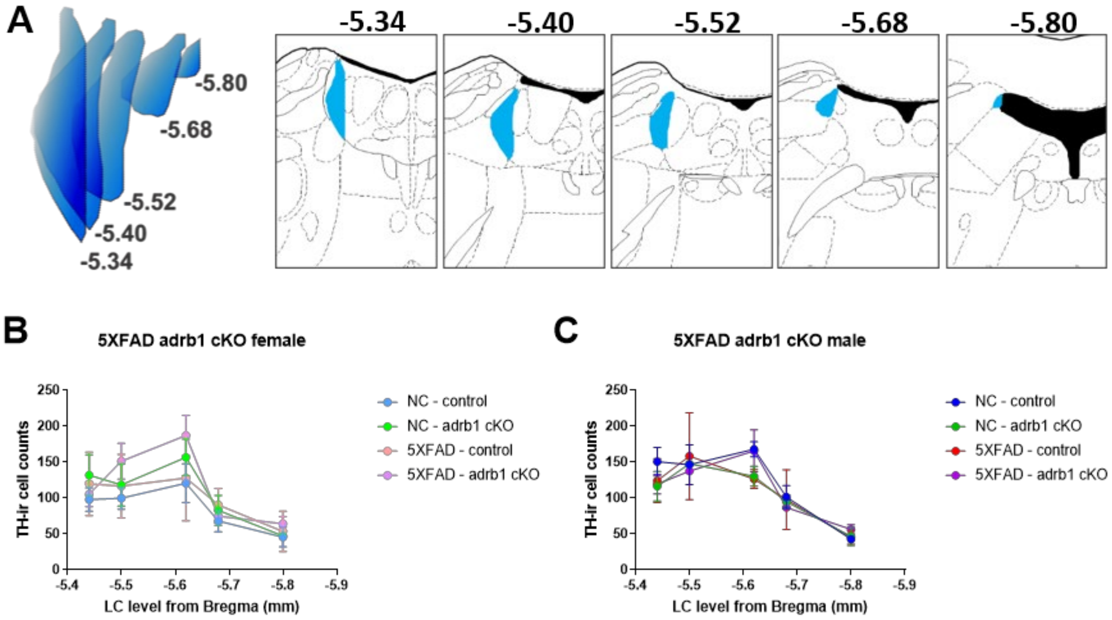

Supplemental Figure S12. Conditional knockdown of adrb1 in mouse microglia does not alter LC neuronal cell counts. **A)** Schematic representation of the rostrocaudal extent of locus coeruleus regional cell quantification with atlas plates. Numbers indicate mm from bregma. Line graphs depict the quantification of LC TH-immunoreactive neurons across the rostrocaudal extent of the LC in **B)** female and **C)** male mice.
