# Supplementary material for: Impact of noradrenergic inhibition on neuroinflammation and pathophysiology in mouse models of Alzheimer’s disease
Source: J Neuroinflammation. 2024 Dec 18;21:322. doi: 10.1186/s12974-024-03306-1 (PMC11657531; doi:10.1186/s12974-024-03306-1)
Supplement: Supplementary file 5 — Supplementary Material 5 [file 12974_2024_3306_MOESM5_ESM.pdf]

Supplemental Methods

RNAScope

RNAScope was performed based off of manufacturers suggested protocol for RNAScope 2.5 HD Duplex Reagent Kit (ACD Bio #322430).

Sample preparation: Fixed hemibrains were sectioned on a cryostat at 40 um and washed in tris-buffered saline (TBS; 10 min x4) and tris-buffered saline with triton (TBST; 10 min). Sections were mounted on superfrost plus slides and dried at RT for 1 hr. Slides were briefly dipped in water x3, dried at RT for 1 hr, and then baked at 60°C for 60 minutes. Sections were then postfixed at 4C for 1 hr in 4% paraformaldehyde prior to dehydration through an ethanol gradient (50%, 70%, 100%, 100%; 5 min each). Slides were dried at 60C for 15 minutes. Sections were quenched with H_2O_2 for 10-20 min at RT, rinsed with water and dried again at 60C for 15 min. Slides were loaded into a rack and submersed into boiling target retrieval solution for 5 min and then washed in distilled water. Slides were rinsed in 100% ETOH 3-5 times and then dried at 60C for 15 minutes. A hydrophobic barrier (ImmEdge Hydrophobic barrier pen, ACD Bio #310018) was drawn around each section and allowed to dry overnight at RT.

Hybridization: RNAScope 2.5 HD Duplex Reagent Kit was used (ACD Bio #322430). Sections were covered in protease plus (2-4 drops) and incubated in the oven for 30 minutes at 40C. Slides were washed in 200 mL distilled water (x2). Excess water was removed from each slide and the slide holder was placed in the humidity control tray. Probe mix (aif1 ACD#319141, adrb1 ACD#449761, adrb2 ACD#449771) (4 drops) was added to cover each section. Tray was placed in oven for 2 hour incubation at 40C. Slides were washed in 200 mL wash buffer with slight agitation for 2 min at RT (x2). Slides were kept overnight in 5X SSC at RT.

Hybridization Amp steps 1-6: Slides were washed with wash buffer (x2). Excess liquid was removed and slide holder was placed into the humidity control tray. AMP 1 (4 drops) were added to cover each section and slides were baked for 30 min at 40C. Slides were washed with wash buffer for 2 min at RT with agitation (x2). This process was repeated with AMP 2 (bake 15 min at 40C), AMP (bake 30 min at 40C), AMP4 (bake for 15 min at 40C), AMP 5 (incubate at RT for 30 min) and AMP 6 (incubate at RT for 15 min). Slides were washed with wash buffer for 2 min at RT with agitation (x2).

Detection of Red Signal: Red-B tube was spun down and combined with Red-A to make Red working solution (1:60 red-B:red-A). Wash was removed from slides and slide holder was placed in the Humidity Control Tray. Red solution (150 uL) was applied to cover each tissue section. Tray was incubated for 10 min at RT. Slides were washed with 1X Wash Buffer for 2 min at RT with slight agitation (x2).

Hybridization AMP steps 7-10: Excess liquid was removed and slide holder was placed into the humidity control tray. AMP 7 (4 drops) was added to cover each section and slides were baked for 15 min at 40C. Slides were washed with wash buffer for 2 min at RT with agitation (x2). This process was repeated with AMP 8 (bake 30 min at 40C), AMP 9 (bake 30 min at 40C), and AMP10 (incubate 15 min at RT). Slides were washed with wash buffer for 2 min at RT with agitation (x2).

Detection of Green Signal: Green-B tube was spun down and combined with Green-A to make Green working solution (1:50 Green-B:Green-A). Wash was removed from slides and slide holder was placed in the Humidity Control Tray. Green solution (150 uL) was applied to cover each tissue section. Tray was

incubated for 10 min at RT. Slides were washed with 1X Wash Buffer for 2 min at RT with slight agitation (x2). Slide were briefly rinsed with water to remove excess wash buffer.

Counterstaining: Sections were counterstained with 50% hematoxylin staining solution for 30 seconds at RT (until sections turn purple). Slide rack was rapidly transferred to a staining dish filled with tap water for up to 30 sec. Slides were then incubated in 0.02% ammonia water (in and out of solution x5) and finally washed in tap water 3-5 times.

Mounting: Slides were dried in 60 C oven for 15-30 min and then cooled for 5 min at RT. Slides were briefly dipped in fresh 100% xylene and coverslipped with vectamount.
